# Characterization of chromosomal architecture in *Arabidopsis* by chromosome conformation capture

**DOI:** 10.1186/gb-2013-14-11-r129

**Published:** 2013-11-24

**Authors:** Stefan Grob, Marc W Schmid, Nathan W Luedtke, Thomas Wicker, Ueli Grossniklaus

**Affiliations:** 1 Institute of Plant Biology and Zürich-Basel Plant Science Center, University of Zürich, Zollikerstrasse 107, CH-8008 Zürich, Switzerland; 2 Institute of Organic Chemistry, University of Zürich, Winterthurerstrasse 190, CH-8057 Zürich, Switzerland

## Abstract

**Background:**

The packaging of long chromatin fibers in the nucleus poses a major challenge, as it must fulfill both physical and functional requirements. Until recently, insights into the chromosomal architecture of plants were mainly provided by cytogenetic studies. Complementary to these analyses, chromosome conformation capture technologies promise to refine and improve our view on chromosomal architecture and to provide a more generalized description of nuclear organization.

**Results:**

Employing circular chromosome conformation capture, this study describes chromosomal architecture in *Arabidopsis* nuclei from a genome-wide perspective. Surprisingly, the linear organization of chromosomes is reflected in the genome-wide interactome. In addition, we study the interplay of the interactome and epigenetic marks and report that the heterochromatic knob on the short arm of chromosome 4 maintains a pericentromere-like interaction profile and interactome despite its euchromatic surrounding.

**Conclusion:**

Despite the extreme condensation that is necessary to pack the chromosomes into the nucleus, the *Arabidopsis* genome appears to be packed in a predictive manner, according to the following criteria: heterochromatin and euchromatin represent two distinct interactomes; interactions between chromosomes correlate with the linear position on the chromosome arm; and distal chromosome regions have a higher potential to interact with other chromosomes.

## Background

In eukaryotic nuclei, chromosomes of considerable length are densely packed into a very small volume. In *Arabidopsis*, chromatin with a total length of about 8 cm has to be packaged into a nucleus of about 70 μm^3^ volume and 5 μm diameter [1,2]. Nonetheless, the extremely dense packaging of chromatin does not lead to a chaotic entanglement of chromatin fibers. Eukaryotes have evolved mechanisms to untangle chromatin and to organize the nucleus into structural domains, facilitating chromosome packaging and, hence, the accessibility of the information stored within chromosomes. Therefore, chromosomal architecture is likely to influence the transcriptional state of a given cell, and might be a major player in the epigenetic regulation of cell fate.

Over the past 15 years, the field of epigenetics has grown rapidly, addressing basic questions about the long-term regulation of genes, and how diverse cell types reach their differentiated states. These studies have provided insights into the mechanisms that enable cells to differentiate into diverse cell types with distinct phenotypes, despite sharing exactly the same genotype.

To date, most of the commonly studied epigenetic processes have been shown to involve covalent modifications of DNA, such as cytosine methylation, modifications of the core histone proteins H3 and H4, and histone variants. Thereby, chromatin can be grouped into activating and repressive chromatin states, defined by their epigenetic landscape. Among the main players are trimethylation of lysine 36 of H3 (H3K36me3) and dimethylation of lysine 4 of H3 (H3K4me2), which act as activating marks, and monomethylation of lysine 27 of H3 (H3K27me1) and dimethylation of lysine 9 of H3 (H3K9me2), which are associated with the repressive state [3-5].

Although studied for over 100 years [6] (for example, with respect to cell division), chromosomal architecture, and thus higher-order chromatin organization, has not been a major focus of epigenetic research. Until recently, the lack of high-resolution techniques made structural studies of the nucleus extremely difficult. Nevertheless, chromatin condensation as seen in heterochromatin, reflecting, chromosomal architecture, could be viewed as the first described epigenetic mark [7,8]. Recently, it became possible to study chromosomal architecture in more detail, on both a global and a local scale, for instance with respect to physical interactions between enhancers and promoters [9,10].

In plants, chromosomal architecture has been studied for many years using cytogenetic techniques and microscopic observations. Early studies allowed the discovery of the basic chromosome conformations, heterochromatin and euchromatin, which were first described in mosses by Emil Heitz as early as 1929 [7]. Most condensed chromatin, or heterochromatin, is associated with centromeric regions. However, large heterochromatic regions outside the pericentromeres were also detected and, because of their microscopic appearance, were termed ‘knobs’. Although first observed and best described in maize [11], knobs were also shown to exist in the model plant *Arabidopsis*, on chromosomes 4 and 5 [12-14]. The heterochromatic knob on the short arm of chromosome 4 (*hk4s*) is derived from an inversion event, which caused a pericentromeric region to lie in a more centrally located region of the chromosome arm. Owing to its length of 750 kb, *hk4s* is easily detectable, and is therefore the best studied knob in *Arabidopsis.* By contrast, the merely 60 kb long knob on chromosome 5 is only poorly described. Despite its central, and therefore euchromatic, position on the chromosome arm, *hk4s* has kept the heterochromatic features of its pericentromeric origin. The knob *h4ks* is characterized by low gene density and an abundance of highly repetitive sequences, such as transposable elements.

To date, two methods have been frequently used to study chromosomal architecture. For microscopic observations, fluorescence *in situ* hybridization (FISH) visualizes chromosomal architecture by detecting specific sections of chromosomes through hybridization with fluorescently labeled probes. Over the past decade, a completely different set of methods has been developed, which are summarized as chromosome conformation capture (abbreviated to 3C) technologies [15,16]. 3C uses formaldehyde cross-linked chromatin that is subsequently digested and religated. This produces circular DNA, comprised of two restriction fragments that were initially in close spatial proximity within the nucleus. The abundance of these circular 3C templates can then be used to calculate interaction frequencies between two given fragments in the genome. In both animal model systems and yeast, various studies have successfully used 3C technologies since the first publication in 2002 [15]. Whereas 3C is used to analyze pair-wise interactions (one specific fragment interacting with another specific fragment; that is, one to one), circular chromosome conformation capture (4C) identifies interactions genome-wide to a viewpoint of interest [17] (that is, one to all). HiC, the most recent 3C technology, facilitates the analysis of genome-wide interactions from all restriction fragments of a genome (that is, all to all) [18].

In the plant field, however, the adoption of these technical advances has been slower, and only a few studies have been performed using 3C technology. A 3C study in maize revealed chromatin looping at the paramutagenic *b1* locus [19], and another recent study showed the importance of local DNA looping for the correct expression of the flowering time regulator locus FLC [20]. Moissiard and colleagues compared global changes in the interactome between mutant *atmorc6* and wild-type plants [21]. However, that study did not focus on a detailed description of the chromosomal architecture of *Arabidopsis* nuclei.

Here, we provide insights into the general architecture of the *Arabidopsis* nucleus, using 4C applied to several viewpoints followed by Illumina sequencing. Our study aimed at characterizing global principles of chromosomal interactions and their correlations with epigenetic marks. Additionally, we found that the heterochromatic knob *hk4s* is characterized by a distinct interactome, which strongly resembles its pericentromeric origin.

## Results

The current knowledge on chromosomal architecture in *Arabidopsis* is largely based on microscopic observations. Therefore, we aimed to gain insights into higher-order chromatin organization based on 4C technology, which promises to complement previously published FISH experiments, and to reveal novel mechanisms governing chromosomal architecture.

**Figure 1:**
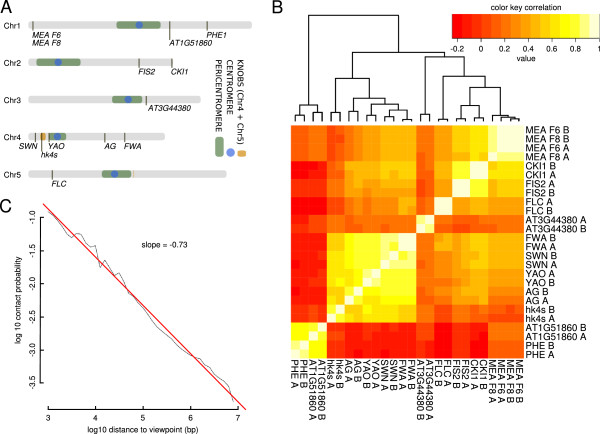
**Primary circular chromosome conformation capture (4C) data analysis. (A)** Schematic representation of the viewpoints chosen for this study. Viewpoints were named according to nearby genes or according to a region of special interest (*hk4s*). **(B)** Cluster analysis representing the reproducibility of biological duplicates. The letters ‘A’ and ‘B ‘at the end of the names indicate biological replicates. **(C)** Power law scaling, indicative of the interaction decay for all viewpoints, across a distance to the viewpoint from 1 kb to 10 Mb.

We performed 4C experiments on aerial tissue of 2-week-old *Arabidopsis* seedlings using thirteen specific restriction fragments (viewpoints) distributed across all five chromosomes (Figure 1A). Employing high-throughput sequencing, 4C technology identifies sequences that physically interact with a given viewpoint. Therefore, the position and number of mapped 4C sequencing reads define the interactome of the given restriction fragment (that is, the viewpoint) in space (position) and in frequency or specificity (number of reads).

To cover a wide distribution of chromosomal interactions, we chose viewpoints that reside in various locations: from pericentromeric, to mid-chromosome arm, to distal positions (Figure 1A).

### Data evaluation reveals robustness of 4C experiments

To obtain the interactome of a given viewpoint, short sequence reads were mapped to restriction fragments, and subsequently merged into sliding windows consisting of 100 *Hind*III restriction fragments. We then assigned *P*-values to each window describing the specificity of the interaction to a given viewpoint. To obtain these *P*-values, read counts of 4C windows were compared with the probabilities of a normal distribution. The parameters of this distribution were calculated using 1,000 sets of windows, each generated by random shuffling of 4C fragments. As chromosome arms differ considerably in their length and, therefore, their DNA amount, we calculated *P*-values individually for each chromosome arm. Windows with *P* ≤ 0.01 where defined as specifically interacting with their corresponding viewpoint and are, hereafter, referred to as ‘preys’.

The mappability of sequencing reads poses a major concern for any genomic study. Owing to the incomplete assembly of centromeric repeats in the *Arabidopsis* reference genome, we excluded regions within 100 kb distance of the centromere. Visual inspection of genomic Illumina sequencing data revealed an even distribution of mapped reads along the remaining chromosome sequence and, therefore, no other major mappability biases were identified.

To assure the reproducibility of this study, 4C experiments were performed in duplicate. Correlations between duplicates and different viewpoints were calculated using the sum of reads per window. Spearman correlation coefficients were high for duplicates (mean ± SD 0.88 ± 0.07), and relatively low for different viewpoints (0.26 ± 0.31). However, interacting viewpoints and viewpoints located in close proximity (see Figure 1A), such as the two viewpoints at the *MEDEA* (*MEA*) locus, had correlation coefficients close to those of replicates of the same viewpoint. Cluster analysis supported these findings (Figure 1B), further demonstrating that viewpoints on the same chromosome arm also show higher correlations with each other than with viewpoints located on other chromosomes arms. Taken together, these analyses reveal the robustness of our data.

To differentiate between random interactions, which are mainly dependent on chromosomal proximity to the viewpoint, and specific interactions, we estimated the genomic distance-dependent decay of the interaction probability on a distance of 1 kb to 10 Mb from the viewpoint. For this, we pooled 4C reads of all viewpoints within the given distance to their viewpoints. Performing linear regression on logarithmized distance and contact probabilities, we calculated a slope of −0.73, that is, the contact probability decays with a power law function of distance^-0.73^ (Figure 1C). This result resembles similar analyses of the *Drosophila* (−0.85) [22] and human (−1.08) [18] genomes.

### *Cis* interactions are enriched within chromosome arms

**Figure 2:**
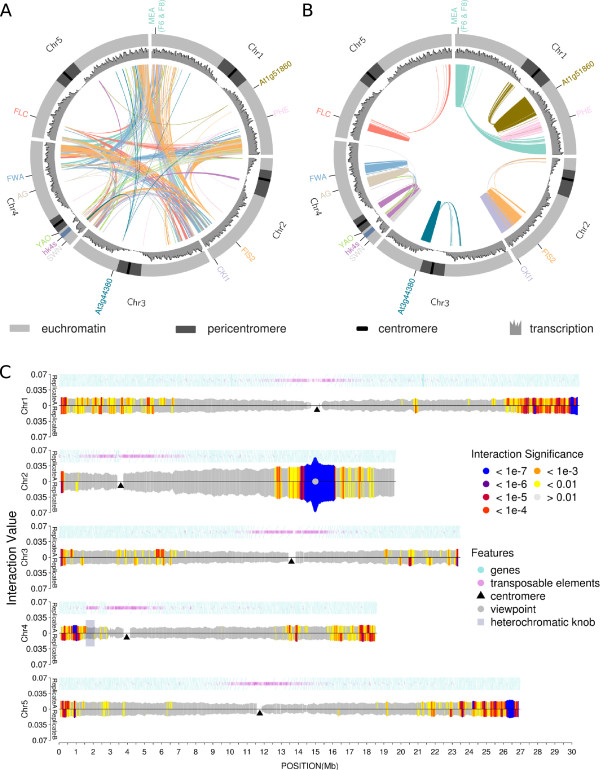
**Summary of circular chromosome conformation capture (4C) interactomes.** Circos plots illustrate the 4C interactome, transcription rate, and chromosomes with euchromatic and centromeric regions. Line color refers to the color of the viewpoint names at the periphery of the Circos plots. Only interactions with a *P* < 10^-3^ are plotted. **(A) ***Trans*- interactions; **(B) ***cis* interactions; **(C)** 4C interactome of viewpoint *FIS2*. Color code refers to significance levels. Gene density (blue circles) and transposable element density (purple circles) are indicated to illustrate the occurrence of heterochromatin and euchromatin. The region covered by the knob *hk4s* is highlighted with a transparent rectangle on the short arm of chromosome 4. Interaction values equal to ∑_i_(log_2_(number of reads in fragment_i_)), where i stands for a fragment within a given window, are scaled to the viewpoint’s total library size.

Because the replicate correlation was high, we pooled replicates for a common representation of the 4C interactome (Figure 2A,B) using the software Circos [23]. Figure 2C illustrates an example of a more detailed representation of 4C interactomes for the *FIS2* viewpoint. All other representations of individual viewpoints are shown in the additional files (see Additional file 1: Figure S1; Additional file 2: Figure S2; Additional file 3: Figure S3; Additional file 4: Figure S4; Additional file 5: Figure S5; Additional file 6: Figure S6; Additional file 7: Figure S7; Additional file 8: Figure S8; Additional file 9: Figure S9; Additional file 10: Figure S10; Additional file 11: Figure S11; Additional file 12: Figure S12; Additional file 13: Figure S13). At first sight, we observed an apparent enrichment in inter-chromosomal interactions of distal regions of chromosomes (Figure 2A). Additionally, intra-chromosomal interactions appeared to be occurring mostly locally around the viewpoint and between the distal regions of the two chromosome arms (Figure 2B and Figure 2C).

Interactions can be categorized into *cis* and *trans* interactions, which require different analysis techniques [24]. *Cis* interactions (Figure 2B) refer to intra-chromosome interactions, whereas *trans* interactions (Figure 2A) are defined as inter-chromosome interactions.

By visual inspection of the interaction frequencies, we observed that local interactions rarely spread across the centromeres, (Figure 2B, Figure 2C; see Additional file 1: Figure S1; Additional file 2: Figure S2; Additional file 3: Figure S3; Additional file 4: Figure S4; Additional file 5: Figure S5; Additional file 6: Figure S6; Additional file 7: Figure S7; Additional file 8: Figure S8; Additional file 9: Figure S9; Additional file 10: Figure S10; Additional file 11: Figure S11; Additional file 12: Figure S12; Additional file 13: Figure S13), indicating that interactions between the two arms of the same chromosome (that is, the inter*-*arm interactions) are distinct from the intra-arm interactions, thus splitting the *cis* interactions into two groups.

**Figure 3:**
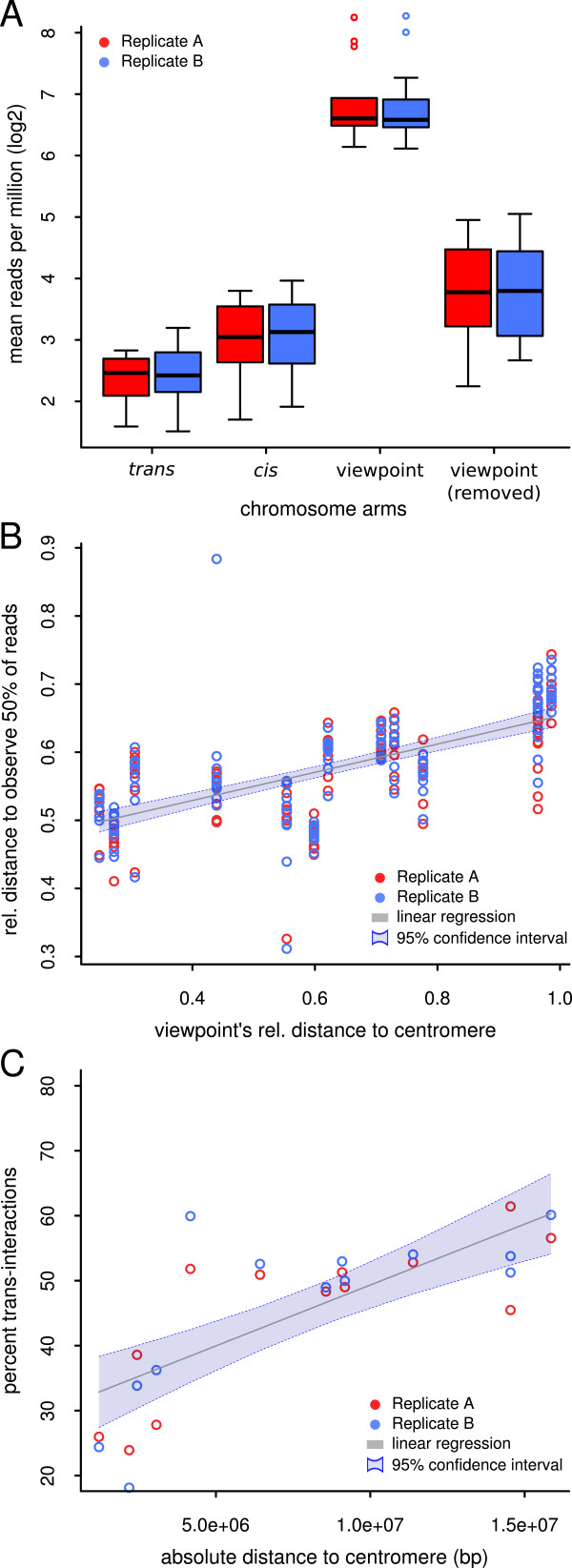
**Physical constraints of chromosomal architecture. (A)** Number of reads per million for four distinct classes of interactomes. Viewpoint: circular chromosome conformation capture (4C) reads that map on the same chromosome arm as the viewpoint. Viewpoint (removed): interactions mapping the viewpoint’s arm, excluding interactions that map within 2 Mb distance on either side of the viewpoint. *Cis*: 4C reads that map to the other arm of the chromosome harboring the viewpoint. *Trans*: 4C reads that map to all other chromosome arms. **(B)** The relative distance to the centromere (0 at the centromere, 1 at the telomere) in which 50% of the 4C reads can be found depends on the relative distance of the viewpoint to the centromere. **(C)** The percentage of 4C reads that can be mapped to *trans* arms was positively correlated with the viewpoint’s absolute distance to the centromere in base pairs (bp). In all parts, red circles represents replicate A, blue represents replicate B.

Therefore, we investigated whether chromosomes, or rather chromosome arms, are the basic unit of nuclear architecture. To answer this question, we calculated the average number of reads per million (RPM) for each chromosome arm, and defined three chromosome arm types: The chromosome arm hosting the viewpoint (viewpoint arm), the other arm on the same chromosome as the viewpoint (*cis* arm), and arms of all other chromosomes (*trans* arms). We observed the highest interaction frequencies and, therefore, the highest mean RPM values within the viewpoint arm (Figure 3A), showing that a high proportion of chromosomal interactions occur within the same arm.

Interactions with *cis* arms were significantly more frequent than those with *trans* arms (Student’s *t*-test, *P* = 0.0135 for replicate A and *P* = 0.0129 for replicate B). However, the differences were small compared with the RPM values for the viewpoint arm and the *cis* arm (Student’s *t*-test, *P* = 1.4 × 10^-13^ for replicate A and *P* = 1.7 × 10^-13^ for replicate B) (Figure 3A). A large proportion of interactions within the viewpoint arm occurred within the close vicinity of the viewpoint itself. To investigate whether long-range interactions also preferentially occur within the viewpoint arm, we excluded regions surrounding the viewpoints by 2 Mb on each side of the viewpoint (Figure 2A). Devoid of the viewpoint region, the RPM values were strongly reduced; however, they were still significantly higher than those of the *cis* arms (Student’s *t*-test, *P* = 0.012 for replicate A and *P* = 0.010 for replicate B).

The difference between the *trans* and *cis* arms appears to be dependent on the distance of the viewpoint from the centromere. Distal viewpoints (for example, *MEA* and *CYTOKININ-INDEPENDENT1* (*CKI1*), see Additional file 1: Figure S1; Additional file 2: Figure S2; Additional file 6: Figure S6) did not appear to interact preferentially with their respective *cis* arm compared with the *trans* arm. This could been observed by comparing the overall interaction values of the viewpoint’s respective *cis* arm compared with the overall interaction values of the *trans* arms. By contrast, viewpoints residing in the vicinity of the centromeres (for example, *YAOZHE* (*YAO*) and *AT3G44380*; see Additional file 7: Figure S7; Additional file 10: Figure S10) exhibited increased *cis* arm interactions compared with *trans* arm interactions and, thus, limited spreading of local interactions across the centromere.

In summary, intra-arm interactions were about ten-fold more frequent than inter-arm interactions, whereas inter-arm and inter-chromosomal interactions differed by about two-fold on average. Therefore, our results show that chromosome arms are the main interaction unit, and that interaction frequencies decrease sharply close to the centromeres.

### Linear position along the chromosome influences the interaction potential of the viewpoint

We found that *trans* interactions could make up to 50% of the total interactome of a given viewpoint. Therefore, we were interested in understanding the mechanisms governing *trans* interactions. Visual inspection of 4C data (Figure 2A, Figure 2C; see Additional file 1: Figure S1; Additional file 2: Figure S2; Additional file 3: Figure S3; Additional file 4: Figure S4; Additional file 5: Figure S5; Additional file 6: Figure S6; Additional file 7: Figure S7; Additional file 8: Figure S8; Additional file 9: Figure S9; Additional file 10: Figure S10; Additional file 11: Figure S11; Additional file 12: Figure S12; Additional file 13: Figure S13) suggested an effect of the viewpoint positions along the chromosome arms on the *trans* interaction frequencies. We hypothesized that chromosomal interactions do not solely reflect specific functions of a given region, but are rather a consequence of physical constraints. To investigate whether the positioning of the viewpoints along the chromosome arm is a major constraint for *trans* interactions, we tested whether regions with similar distance to the centromeres are more likely to interact.

We calculated the relative distance to the centromeres, where 50% (dist_0.5_) of all 4C reads could be found. As a considerable proportion of all interactions could be found surrounding the viewpoint and would therefore distort the analysis, we excluded the viewpoint arm. A significant correlation between dist_0.5_ and the relative distance of the viewpoint to the centromere could be observed (Spearman correlation coefficient = 0.722; linear model *P* = 3.4 × 10^-28^) (Figure 3B). This suggests that regions with a similar relative distance to their corresponding centromeres are likely to co-localize with each other in the three-dimensional space of the nucleus. This observation was most pronounced in distal regions; however, it was also observable in regions in proximity to the pericentromeres.

### Distal chromosomal regions show an increased *trans* interaction potential

We hypothesized that the flexibility of a chromosome arm is a major physical constraint influencing the interaction potential of a viewpoint. Assuming that centromeres act as chromosomal anchors, distal regions of chromosome arms should exhibit a higher flexibility than regions close to the centromere [25-28]. Hence, we predicted that distal viewpoints should exhibit an increased *trans* interaction potential.

Therefore, we tested the correlation between the absolute distance of the viewpoint to the centromere and the reads per kilobase per million (RPKM) of 4C reads found in *trans* (including the *cis* arm) (Figure 3C). Distal viewpoints were shown to interact more frequently with regions in *trans* than did viewpoints residing closer to the centromere (Spearman correlation coefficient = 0.774, linear model *P* = 10^-5^) (Figure 3C).

These results indicate that the localization of a viewpoint along the chromosome arm significantly influences its interaction pattern.

### Principal component analysis showed a correlation between the epigenetic landscape and the interactome

The interplay of epigenetic marks, such as histone modifications, and physical interactions of two sequences were previously shown to be important for stringent gene regulation [20,22,29,30]. Therefore, we investigated whether specific epigenetic marks can be correlated with long-range interactions.

We obtained previously published histone modification data [31], specifically H3K4me2, H3K4me3, H3K9me2, H3K27me1, H3K27me3, H3K36me2, H3K36me3, H3K9ac, and H3K18ac. From the same dataset, we included transcriptome, histone H3 occupancy, and genomic DNA control data. Additionally, we obtained publically available CG, CHH, and CHG DNA methylation data [32]. Because data obtained from chromatin immunoprecipitation (ChIP) for histone modifications cannot be directly compared with 4C data due to the different scaling of the two datasets [24], we calculated density values of each epigenetic feature within 4C windows. We analyzed the epigenetic modification densities (EMDs) as the sum of nucleotides covered by at least one uniquely alignable short sequence, divided by the total number of nucleotides for each individual 4C restriction fragment (that is, the length of the restriction fragment). Subsequently, the mean for each window was calculated. To adjust the scale of the 4C data to the EMDs, we chose a window size of 25 fragments, which still conferred satisfactory reproducibility between replicates. 4C windows were categorized into prey regions (windows that show an interaction probability of ≤0.01) and randomly chosen control regions.

**Figure 4:**
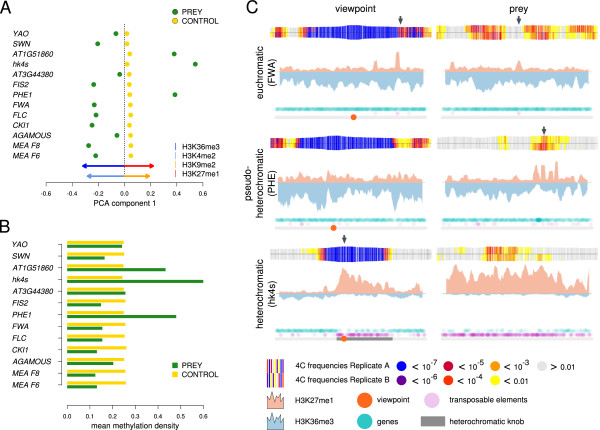
**Crosstalk of epigenome and interactome. (A)** Principal component analysis (PCA) using mean epigenetic modification densities (EMDs) of control and prey regions for each viewpoint. EMDs included in the PCA were: H3K4me2, H3K4me3, H3K9me2, H3K27me1, H3K27me3, H3K36me2, H3K36me3, H3K9ac, and H3K18ac. Colored arrows represent the two highest contributing EMDs to the variance of the first component in positive and negative direction, respectively. Note that the first principal component explains almost all the variance (97%), and therefore, this was the only component plotted. Prey regions are represented by green dots, control regions by yellow dots. **(B)** Mean CG methylation densities of prey and control regions for individual viewpoints. The mean was calculated across 1000 times randomly sampled 50 prey and 50 control regions, respectively. Green bars represent preys and yellow bars represent controls. **(C)** Examples of the interactome-epigenome interplay for three different viewpoints and one of their corresponding prey regions. Top track: log summed 4C reads per window (100 fragments, starting every fragment). 4C reads of replicate A are plotted in the positive intercept, and 4C reads of replicate B are plotted in the negative intercept. Middle Track: EMD of the highest contributing factors of the PCA in positive and negative direction, respectively. In order to achieve comparable representation of H3K36me3 and H3K27me1 densities, the density of every window (25 fragments, starting every 5 fragments) was divided by the mean density of each histone modification. Arrowheads point at regions where the 4C interactome and local EMD peaks appeared to correlate. *FWA*: viewpoint on chromosome 4, 12 to 14 Mb; prey on chromosome 5, 23 to 25 Mb. *PHE*: viewpoint on chromosome 1, 23.5 to 25.5 Mb; prey on chromosome 1, 20 to 22 Mb. *hk4s*: viewpoint on chromosome 4, 0.8 to 2.8 Mb; prey on chromosome 2, 4 to 6 Mb.

If specific histone modifications or sets of histone modifications are associated with an interaction pair, it could be assumed that prey regions of a given viewpoint would share a common epigenetic environment, reflected by a particular composition of the EMDs. To elucidate how histone modifications are related to the interactome, we performed principal component analysis (PCA) (Figure 4A). For each viewpoint, the mean EMDs (selecting only histone modification data) of prey and control regions were calculated and included in the PCA. As the first principal component was found to explain 97% of the total variation, it was the only component used for further analyses.

Two opposing groups of EMDs, H3K36me3/H3K4me2 and H3K27me1/H3K9me2, were found to be the major contributors to the first principal component of the PCA (Figure 4A, arrows). Closer observation of three viewpoint/prey pairs revealed how EMDs and interaction frequencies are coupled (Figure 4C). Euchromatic viewpoints, such as *FLOWERING WAGENINGEN* (*FWA*) (Figure 4C, top row), which are characterized by low levels of H3K27me1 and enrichment of H3K36me3, preferentially interacted with regions of a similar EMD pattern. This is evident from the increased H3K36me3 levels surrounding the region of high interaction frequencies and local peaks of H3K27me1 enrichment, coinciding with a significant drop in interaction frequencies (Figure 4C, top row, right panel). By contrast, heterochromatic viewpoints (Figure 4C, middle and bottom rows), which are characterized by the inverse EMD composition, preferentially interacted with regions exhibiting low H3K36me3 and high H3K27me1 levels. For example, local enrichment of H3K27me1 coincided with increased interaction frequencies to *PHE1* (Figure 4C, middle row, right panel). Moreover, the asymmetric local interactions surrounding *hk4s* appeared to be reflected by the asymmetric distribution of H3K27me1 (Figure 4C, bottom row, left panel).

Additionally, we performed PCA separately for individual viewpoints (see Additional file 14: Figure S15). Although the same EMDs could be identified as major factors for most viewpoints, the first component of the PCA was less dominant, indicating a more complex collaboration of factors separating control regions from prey regions. Furthermore, various viewpoints did not show a very clear separation of prey and control regions. Interestingly, this was most evident for viewpoints whose preys are associated with heterochromatic marks (*PHERES1* (*PHE1*), *hk4s*, *AT1G51860*) (see Additional file 14: Figure S15).

To address the individual contribution of epigenetic marks to the interactome, we performed a test based on a modified Gene Set Enrichment Analysis (GSEA) [33]. In summary, we tested whether prey regions would show a non-random distribution in their EMD profiles (see Materials and Methods for a detailed description). The obtained empirical *P*-values are indicative of the likelihood of a random set of regions to show a similar distribution of EMD values as the tested prey regions (Table 1).

**Table 1:** Analysis of the epigenetic landscape

**Genomic feature**	** P-value**^**a**^	
	**Permutation test**	**GSEA-like test**
H3	0.1013	0.0779
H3K18ac^b^	**0.0335**	**0.0178**
H3K27me1^b^	**0.0249**	**0.0084**
H3K27me3	0.3355	0.099
H3K36me2^b^	**0.0033**	**0.0051**
H3K36me3^b^	**0.0033**	**0.0054**
H3K4me2^b^	**0.0033**	**0.0051**
H3K4me3^b^	**0.0037**	**0.0051**
H3K9ac^b^	**0.0033**	**0.0051**
H3K9me2^b^	**0.0325**	**0.0057**
Transcription^b^	**0.0033**	**0.0054**
CG methylation replicate 1^b^	**0.0065**	**0.0054**
CHG methylation replicate 1^b^	**0.0083**	**0.0051**
CHH methylation replicate 1^b^	**0.0083**	**0.0051**
CG methylation replicate 2^b^	**0.0083**	**0.0054**
CHG methylation replicate 2^b^	**0.0087**	**0.0051**
CHH methylation replcate 2^b^	**0.0083**	**0.0051**
Genomic DNA	0.0871	0.056

^a^Table contains adjusted *P-*values (false discovery rate; FDR (Benjamini-Hochberg)) for genomic features tested with a permutation test or a Gene Set Enrichment Analysis (GSEA)-like algorithm.

^b^Genomic features differing significantly between prey and control regions (α = 0.05).

To independently investigate whether control and prey regions differ significantly for individual epigenetic features, we developed a permutation test. In the first step, we calculated for each viewpoint the mean density for each epigenetic feature (Figure 4B and Additional file 15: Figure S16). Epigenetic features that coincide with the occurrence of heterochromatin and euchromatin, such as DNA methylation, clearly split the viewpoints into two groups. Whereas viewpoints such as *PHE1*, *AT1G51860*, and *hk4s* had high methylation levels in their prey regions and low methylation levels in control regions, viewpoints that occur in euchromatin showed an inverse pattern. Similar patterning was also detectable for other epigenetic modifications (Figure 4B; see Additional file 15: Figure S16).

The inverse patterning of the epigenetic landscape between different viewpoints made it difficult to perform statistical tests using EMD values directly. Therefore, we calculated the absolute difference in the density of the epigenetic features density between control and prey regions. In essence, we tested whether the absolute difference in EMD values between prey and control regions were significantly different from the absolute difference between two sets of randomly selected regions. As a test set, we shuffled the 50 prey and 50 control regions into two randomized groups. As for the prey and control regions, we then calculated means and subsequently absolute differences between the two randomized groups. By repeating the permutations 1,000 times, we obtained a distribution of absolute differences between the two randomized groups for each epigenetic feature. This allowed us to calculate empirical *P*-values, which describe the chance that two randomly selected regions would differ more in their EMD setup than would prey and control regions (Table 1).

In line with the previously performed PCA, both tests revealed that the densities of most epigenetic features differed significantly between control and prey regions (Table 1). Histone H3 occupancy, however, did not differ significantly between the two groups, indicating that histone density itself does not correlate with a viewpoint’s interactome. Additionally, no significant difference in genomic control data could be observed, rendering possible sequencing and alignment biases of the analyzed EMD dataset unlikely.

In summary, we conclude that the epigenetic landscape coincides with the interactome. This is mainly reflected by distinct euchromatic and heterochromatic interactomes.

### The heterochromatic knob evades its euchromatic environment

**Figure 5:**
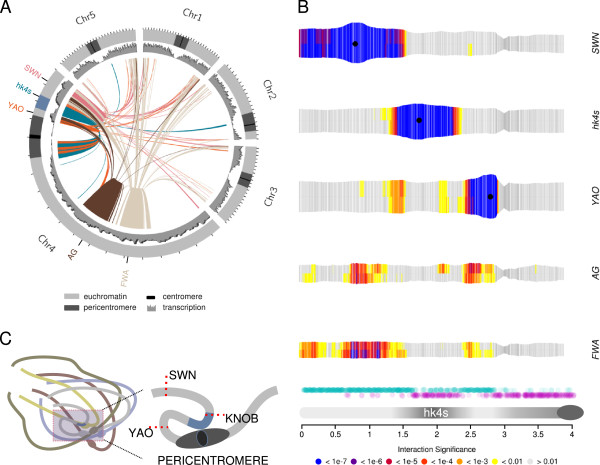
**Interactome of the knob *hk4s. *(A)** Circos plot illustrating all *cis* and *trans* interactions of viewpoints located on chromosome 4. Only interactions with *P* ≤ 10^-4^ were considered. Line color corresponds to the color of the viewpoints name indicated at the periphery of the plot. Chromosomes are not drawn to scale. **(B)** Representation of interaction frequencies for viewpoints situated on chromosome 4. Note that only the region up to 4 Mb is plotted, therefore, viewpoints *AG* and *FWA* cannot be seen. Black dots show positions of viewpoints; turquoise dots, genes; violet dots, transposable elements; light grey, euchromatic chromosomal segment; dark grey, heterochromatic chromosomal segments; dark grey ellipse, centromere. **(C)** Model of a potential mid-range chromosomal loop, connecting *hk4s* with the centromere of chromosome 4.

Analyzing the read numbers of a first set of 4C viewpoints, we consistently observed a drop in read numbers for a region situated in the center of the short arm of chromosome 4 (Figure 5B; see Additional file 1: Figure S1; Additional file 2: Figure S2; Additional file 3: Figure S3; Additional file 4: Figure S4; Additional file 5: Figure S5; Additional file 6: Figure S6; Additional file 7: Figure S7; Additional file 8: Figure S8; Additional file 9: Figure S9; Additional file 10: Figure S10; Additional file 11: Figure S11; Additional file 12: Figure S12; Additional file 13: Figure S13). Unexpectedly, this drop in interaction frequency was observed irrespective of the location of the viewpoint. Additionally, we did not observe this drop with visual inspection of genomic sequencing data, implying no mappability bias. Therefore, we hypothesized that global constraints of chromosomal architecture govern genome-wide interactions with this region.

Exploring the region in more detail, we found that it corresponds to the heterochromatic knob (*hk4s*), which is cytogenetically detectable and has been described previously [12,34] (see Additional file 9: Figure S9).

To analyze the implications of *hk4s* on chromosomal architecture in more detail, we designed three additional 4C assays. We set a viewpoint within *hk4s* and two viewpoints flanking *hk4s* in a more distal region (*SWINGER* (*SWN*)) and a more proximal region (*YAO*) of the short arm of chromosome 4. As the flanking viewpoints were set relatively close to *hk4s*, we expected increased frequencies of interactions within the knob and the viewpoints, owing to the previously observed local enrichment of interactions surrounding the viewpoints. However, the local interaction frequency of both neighboring viewpoints dropped sharply on the borders of *hk4s* (Figure 5A, Figure 5B; see Additional file 8: Figure S8; Additional file 9: Figure S9; Additional file 10: Figure S10). *YAO* (coordinate at 2.75 Mb) is situated adjacent to the border of the pericentromere (coordinates 2.78 to 5.15 Mb) [3]. Interestingly, the local interaction pattern appears to be asymmetric. We observed a loss of specific interactions not only along the boundary to the knob but also along the much closer border of the pericentromeric region (Figure 5B; see Additional file 10: Figure S10). The defined sharp boundaries for local *YAO* interactions resembled the interaction pattern of *hk4s*. Whereas *YAO* resides in euchromatin surrounded by heterochromatin, *hk4s* can be viewed as its counterpart, residing in heterochromatin but surrounded by euchromatin (Figure 5B).

Regions situated on the long arm of chromosome 4 (*AGAMOUS* (*AG*) and *FWA*) interacted strongly with regions surrounding *hk4s,* including *YAO*, but not with *hk4s* itself (Figure 5B; see Additional files 11: Figure S11; Additional file 12: Figure S12), resembling the sharp drop in the interaction frequencies of *SWN* and *YAO* (Figure 5A, Figure 5B; see Additional file 8: Figure S8; Additional file 9: Figure S9; Additional file 10: Figure S10).

Consistent with observations for the two flanking viewpoints, the significant local interaction frequencies of the viewpoint set in the center of *hk4s* were limited by the borders of the knob. Additionally, we observed strong interactions of *hk4s* with the pericentromeric regions of chromosome 4 and with the pericentromeres of other chromosomes (Figure 5A). The apparent absence of specific interactions between *hk4s* and the pericentromere of the short arm of chromosome 4 is likely to be an artifact of the method used to assign *P*-values. Indeed, as *P*-values were calculated for individual chromosome arms, the high number of reads covering the viewpoint itself masks other regions on the same chromosome from being associated with low *P*-values.

## Discussion

### Replication and the choice of appropriate window size are key to ensuring robustness of 4C

Based on a correlation analysis of biological replicates, we show that 4C interaction profiles in *Arabidopsis* can be reproducibly obtained. However, reproducibility is dependent on the window size chosen. As chromosomal interactions are dynamic and partly stochastic, one single restriction fragment of two replicates can vary considerably in read number. Taking windows consisting of several fragments into account can balance this variation. As we were mainly interested in the global architecture of the *Arabidopsis* nucleus, we chose window sizes of up to 100 restriction fragments. However, the resolution for studying short-range interactions is decreased by increasing the window size. Whereas 4C is well suited to study mid-range and long-range interactions in *Arabidopsis*, it is not necessarily the method of choice to study short-range interactions (for example, promoter/enhancer interactions). Regulatory sequences that are presumably involved in short-range interactions, such as chromatin loops, are often separated by less than a few kb. They are, therefore, difficult to analyze using 3C technologies, which rely on a sufficient number of restriction sites between the two regions of interest to confer satisfactory resolution.

### *Arabidopsis* and *Drosophila* show comparable chromatin compaction and genome size

The interaction decay exponent describes the slope with which the interaction probability decays from the viewpoint. Therefore, it can provide an approximation of regional chromosomal compaction. Theoretically, a steeper slope indicates decreased flexibility of a given viewpoint, as distant regions are less likely to interact with it. Decreased flexibility can be interpreted as higher local chromatin compaction. *Drosophila* and *Arabidopsis* are similar with respect to chromosome number, genome size, total number of genes, and nuclear volume [1,35]. These characteristics could lead to similar constraints of chromosomal architecture. The interaction decay exponent determined in this study (−0.73) is close to that described earlier for *Drosophila* (−0.85) [22]. Interestingly, the interaction decay exponent in human nuclei is lower (−1.08), implying higher local compaction [18]. This observation is consistent with the physical characteristics of human nuclei compared with those in *Arabidopsis* and *Drosophila*. Although varying considerably, human nuclei show a lower volume/DNA ratio than the nuclei in *Drosophila* and *Arabidopsis*, indicating a higher global chromatin compaction [35]. It is important to mention, however, that interaction decay exponents cannot be compared very easily between different studies, as the calculated exponents of the power law scaling depend on the range of distances used for calculations. However, which scale best describes an overall distance-dependent interaction decay is a matter of debate. Additionally, the slope with which interactions decay was previously shown to vary between domains with different epigenetic landscapes [18,22]. We observed a variation in interaction decay exponents between the different viewpoints, from −0.56 to −0.96 (see Additional file 16: Figure S14). However, we could not explain these differences, either by the positional or by the epigenetic environment of a given viewpoint. Therefore, the global distance-dependent interaction decay does not necessarily add to the understanding of how interaction frequencies decrease with distance from an individual viewpoint.

How and whether global nuclear compaction and interaction probability decay really correlate is not entirely clear. An exploration of the *Arabidopsis linc1,linc2* double mutant could possibly answer this question, as these plants were reported to exhibit increased DNA density compared with wild-type plants [1].

### 4C results refine the view on general chromosomal architecture in *Arabidopsis*

The investigation of general features of chromosomal architecture in this study is consistent with previous findings studying *Arabidopsis* nuclei using cytogenetic methods [27,36]. However, 4C technology enables us to generate genome-wide interaction maps for various viewpoints and, hence, does not depend on a pair-wise analysis of two interacting sequences. This greatly adds to our understanding of general constraints on chromosomal architecture.

Basic interaction units appear to be defined as chromosome arms, with centromeres acting as a boundary. These findings are in agreement with an earlier study by Schubert and colleagues, reporting that chromosome arms are localized in distinct territories, as evidenced by FISH on *Arabidopsis* nuclei [36]. However, whether centromeres always act as strict boundaries cannot be conclusively answered, as the boundary effect of centromeres is likely to vary between the different chromosomes.

We observed a strong influence of the chromosomal location of a viewpoint on its interaction potential. Remarkably, the linear organization of chromosomes was reflected in the overall interaction potential of a given viewpoint, despite the dense packaging of the genome in the nucleus.

We propose that centromeres anchor the chromosomes in the nucleus, thereby allowing chromosome arms to protrude inside the nuclear volume [25-28]. The flexibility of chromosome arms thus increases with their length, allowing distant regions to interact more frequently in *trans* than more centrally located regions. Our hypothesis is supported by strong evidence for clustering of centromeres and their adherence to the nuclear matrix in different model organisms [37-39]. Taken together, these findings may explain why regions with a similar distance to the centromeres, which act as anchor points, preferentially interact with each other.

We also observed significant inter-telomeric interactions. A high interaction frequency of (sub-)telomeric regions in *Arabidopsis* was recently also shown by FISH [36]. In addition, previously published HiC data suggest increased interaction frequencies between telomeres [21,38]. By contrast, telomeres and centromeres do not interact, indicating a strict separation of these two key organizational elements of *Arabidopsis* chromosomes. These findings are in line with previous studies, and may be explained by the nucleolar localization of telomeres [27,40].

Remarkably, in *Drosophila*, long-range interactions seem to occur nearly exclusively within the viewpoint’s chromosomal arm [30]; however, in the present study, up to 50% of all interactions were found to be outside this region*.* Whether this difference from *Drosophila* holds biological meaning is unclear. The presence of a higher number of individual cell types in the sample could theoretically increase the number of observable interactions, and result in a more complex interactome of a given viewpoint. Such increased complexity could thereby lead to an increased number of *trans* interactions. However, we do not estimate the number of cell types to be significantly different between the present study and the report by Tolhuis and colleagues, in which 4C was performed on *Drosophila* larval brain tissue [30], as the aerial seedling tissue used in our study is predominantly composed of mesophyll cells. The phase of the cell cycle might be a more important confounding factor. Over a cell cycle, chromosomal architecture changes dramatically. Cells of *Arabidopsis* seedlings divide at high frequency, leading to a rather short time period in which cells reside in interphase. Therefore, the proportion of cells in specific stages of the cell cycle could be a major factor influencing the (average) chromosomal conformation of a population of cells.

### The interactome of a viewpoint is reflected in its epigenetic landscape

PCA revealed two distinct groups of prey regions, which could be discriminated mainly by the level of H3K36me3/H3K4me2 and H3K27me1/H3K9me2 densities. Interestingly, these histone modifications are commonly attributed to euchromatin or heterochromatin, respectively [31]. Furthermore, the heterochromatic pair H3K27me1/H3K9me2 is described to be the major component of ‘chromatin state 3’, which is mainly associated with transposable elements, as previously reported by Roudier and colleagues, whereas the pair H3K36me3/H3K4me2 primarily contributes to ‘chromatin state 1’, associated with active genes [3]. Filion and colleagues describe five distinct chromatin types in *Drosophila*, distinguished by the composition of proteins adhering to the DNA. H3K4me2 was shown to be most abundant in ‘red chromatin’, which represents one of two euchromatic chromatin states, whereas H3K9me2 is enriched in ‘green chromatin’, which can best be described as the classic heterochromatin of pericentromeric regions [4]. As anticipated by previous cytological studies of *Arabidopsis* nuclei, the interactome obtained by 3C technologies can be separated into two distinct domains, correlating with both the epigenetic and the cytogenetic definition of heterochromatin and euchromatin. Interestingly, this distinction is not only confined to *cis* interactions but can also be observed at the level of the whole genome. In addition, we suggest a further discrimination of heterochromatic interactions. The purely heterochromatic viewpoint *hk4s* predominantly interacts with visible heterochromatin such as the pericentromeric regions. *PHE1*, which shows moderate H3K27me1 enrichment surrounding the viewpoint, interacts predominantly with heterochromatic islands within otherwise euchromatic regions (Figure 2, Figure 4C; see Additional file 4: Figure S4).

Previous work in *Arabidopsis* has shown that homologous pairing is decreased in hypomethylation mutants [41], indicating a role for cytosine methylation in long-range interactions. We observed significant differences between control and prey regions with respect to their CG, CHH, and CHG methylation densities. Additionally, transcription rates exhibited significant differences between prey and control regions. Whether transcriptionally active genes interact with each other is not clear, as the genes residing in our viewpoints were not evenly balanced with regard to their transcriptional state (active versus silenced), rendering them inappropriate for statistical analysis.

Taking these results together, we conclude that interactomes share a common epigenetic landscape, leading to distinguishable heterochromatic and euchromatic interactomes. However, it is not clear to what extent individual epigenetic modifications influence the interactome, and to what extent the epigenetic landscape is the cause or consequence of a given interactome.

### The knob *hk4s*: exception or rule?

Finally, the knob *hk4s* appears as an exceptional feature within the *Arabidopsis* nuclear landscape, as it interacts predominantly with pericentromeric regions. We think that *hk4s* represents the exception that proves the rule because its interactome reflects the pericentromeric origin of *hk4s*, which arose by an inversion that placed a pericentromeric region into the center of the chromosome arm. As discussed above, heterochromatic regions form a distinct interactome, in which heterochromatic islands that reside in an euchromatic environment are included. Figure 5C illustrates a model suggesting overall chromosomal architecture and chromosomal looping of *hk4s* to the clustered centromeres. Our results indicate that the knob *hk4s* acts as an interaction insulator for its neighboring regions, and conserves its pericentromeric origin with respect to its interaction frequencies.

To date, neither a functional role as a (neo)centromere nor an association with the nuclear matrix has been reported for *hk4s.* However, the specific interaction of *hk4s* with centromeres could raise speculation concerning the functional role of *hk4s* in the nucleus. The specificity of a given region to function as a centromere is surprisingly flexible. Previous reports show that in maize, centromere identity is not irreversibly defined. Wolfgruber and colleagues demonstrated that the centromere of maize chromosome 5 has moved to a new location, due to the invasion of non-centromeric retrotransposons, splitting the centromere into two. Consequently, one of the two cleavage products lost its association with histone CenH3, which defines centromeres epigenetically by replacing the regular histone H3 protein [42]. In maize, centromere identity correlates with the abundance of centromeric retrotransposons [43], which specifically invade centromeric regions. Nevertheless, centromere identity appears to be mainly controlled epigenetically and not by DNA sequence [44,45]. However, previous reports show that that histone CenH3 accumulation defines the functional centromere in *Arabidopsis* and that CenH3 is predominantly associated with the 178 bp centromeric repeats [46,47]. As the knob *hk4s* lacks the centromeric 178 bp repeats and is thought to originate from a pericentromic region, which is not associated with CenH3, we conclude that *hk4s* is mainly involved in heterochromatin formation, and that *hk4s* is unlikely to play a role as a (neo)centromere.

## Conclusions

Centromeres are key elements for chromosomal organization, as the position relative to the centromere strongly influences the interactome of a chromosomal region. We propose that the length of chromosome arms limits the mobility with which a region can traverse through the nuclear space and, therefore, influences the interaction potential in *trans*. Another hallmark of chromosomal architecture in *Arabidopsis* nuclei is the separation of two seemingly distinct interactomes, strongly correlating with visible heterochromatin and euchromatin. Interestingly, heterochromatic islands are partly able to evade their euchromatic context. The epigenetic landscapes of the heterochromatic and euchromatic interactome are clearly distinguishable. Therefore, histone modifications, which were previously described to be characteristic of chromatin states, may also be predictive for the interaction potential of a given chromosomal region.

## Materials and methods

### Nuclei extraction and 4C sample preparation

Seedlings of *Arabidopis thaliana* (L.) Heynh*,* accession Columbia (Col-0), were grown for 14 days on MS plates (4.3 g/l Murashige and Skoog salt (Carolina Biological Supply Company, Burlington, North Carolina, USA), 10 g/l sucrose (Applichem GmbH, Darmstadt, Germany), 7 g/l PHYTAGAR (Life Technologies Europe, Zug, Switzerland), pH5.6). Aerial tissue of seedlings was collected (approximately 10 g per sample), and distributed evenly between four conical 50 ml tubes. Under vacuum, the seedlings were incubated for 1 hour at room temperature in 15 ml freshly prepared nuclei isolation buffer (NIB: 20 mmol/l Hepes (pH8), 250 mmol/l sucrose, 1 mmol/l MgCl_2_, 5 mmol/l KCl, 40% (v/v) glycerol, 0.25% (v/v) Triton X-100, 0.1 mmol/l phenylmethanesulfonylfluoride (PMSF), 0.1% (v/v) 2-mercaptoethanol) and 15 ml 4% formaldehyde solution, then 1.9 ml of 2 mol/l glycine was added to quench the formaldehyde, and the mixture was incubated for another 5 minutes under vacuum. The seedlings were snap-frozen in liquid nitrogen, and ground to a fine powder. The powder from two initial tubes was pooled and suspended in 10 ml NIB, with added protease inhibitor (Complete Protease Inhibitor Tablets; Roche, Basel, Switzerland; two tablets in 150 ml NIB). The suspension was filtered twice through Miracloth (Calbiochem/EMD Milipore, Darmstadt, Germany) adding an additional 10 ml NIB. The filtered nuclei suspension was spun for 15 minutes at 4°C and 3000×*g*. The supernatant was discarded, and the pellet was resuspended in 4 ml NIB and transferred to two 1.5 ml reaction tubes. After the tubes were spun for 5 minutes at 4°C and 1900×*g*, the supernatant was removed, and the pellet was resuspended in 1 ml NIB, followed by centrifugation under the above conditions. This step was repeated twice. Then, the nuclei were washed twice with 1.2 × NEB buffer 4 (New England Biolabs, Ipswich, MA, USA) (10 × NEB buffer 4: 50 mmol/l potassium acetate, 20 mmol/l Tris acetate, 10 mmol/l magnesium acetate, 1 mmol/l dithiothreitol (DTT)), using the centrifugation conditions described above. The nuclei were finally resuspended in 500 ml 1.2 × NEB buffer 4, with 5 μl of 20% SDS added. The samples were incubated for 40 minutes at 65°C, followed by 20 minutes at 37°C under constant shaking, then 50 μl of 20% Triton X-100 were added. The mixture was incubated for 1 hour at 37°C under constant shaking, then 60 μl of sample was removed as a pre-digestion control.

For digestion 15 μl 10 × NEB buffer 4 and 115 μl H_2_0 were added to the samples, and digestion was started using 100 U of *Hind*III restriction enzyme (New England Biolabs). After 3 hours of incubation at 37°C, 200 U of *Hind*III were added, followed by overnight incubation at 37°C. Next morning 100 U of *Hind*III were added, and samples were incubated for a final 2 hours. An aliquot (80 μl) of the sample was transferred to a fresh tube, and kept aside as a post-digestion control. To inactivate *Hind*III, 20 μl 20% SDS were added, and samples were incubated at 65°C for 25 minutes under constant shaking. Samples were transferred to 15 ml conical tubes, and 700 μl of 10× ligation buffer (0.5 mol/l Tris-Cl, 0.1 mol/l MgCl_2_, 0.1 mol/l DTT, pH 7.5), 375 μl of 20% Triton X-100, and H_2_O to a final volume of 7 ml was added, followed by 1 hour of incubation at 37°C under constant shaking.

Ligation was performed by adding 70 μl of 100 mmol/l ATP (Roche) and 50 Weiss Units (WU) of DNA Ligase (Fermentas/ThermoFisher, Waltham, USA). The sample was incubated for 5 hours at 16°C. During incubation, additional 10 WU of DNA ligase were added. Following ligation, 30 μl 10 mg/ml proteinase K (Qbiogene; MP Biomedicals, Santa Ana, CA, USA) were added, and the sample was incubated overnight at 65°C. Next morning, 30 μl of 10 mg/ml RNase A (Roche) were added, and the sample was incubated for 30 minutes at 37°C.

The DNA was purified by two chloroform:phenol extractions, followed by ethanol precipitation using 1 ml 3 mol/l sodium acetate, 7 ml H_2_O and 25 μl glycogen, taken up to a final volume of 50 ml with ice-cold ethanol. The mixture was kept overnight at −80°C. The pellet was finally resuspended in 150 μl H_2_O.

Pre-digestion control, post-digestion control, and the final 3C sample (120 ng of DNA each) were analyzed on 1.5% agarose gels. Samples with satisfactory digestion were then pooled to proceed further.

The 3C samples were digested with a final quantity of 0.2 U/μl of the secondary restriction enzymes *Dpn*II or *Nla*III, respectively (New England Biolabs). The 4C digested samples were analyzed on an agarose gel. For the 4C ligation, 700 μl of T4 Ligase Buffer (Fermentas/ThermoFisher), 70 μl 100 mmol/l ATP, and 50 WU of DNA Ligase (Fermentas/ThermoFisher), were taken up to 7 ml with H_2_O; this mixture was added to the samples, and the ligation reaction was incubated for 5 hours at 16°C. Finally, the samples were purified by phenol:chloroform extraction, followed by ethanol precipitation, and stored at −20°C.

For each viewpoint, 16 PCRs (for detailed PCR conditions and primer sequences, see Additional file 17: Table S1) were set up, using 30 ng of 4C template for each reaction. For ease of later Illumina library preparation, primers of a subset of samples were designed with an Illumina sequencing adapter tail (batch 1: *MEA F6*, *MEA F8*, *PHE*, *FIS2*, *CKI1*, *FWA*, *AG, FLC*). For all other samples (batch 2: *AT1G51860*, *AT3G44380*, *SWN*, *hk4s*, *YAO*), Illumina sequencing adapters were ligated later in the library preparation process.

An aliquot of each PCR product was analyzed on an agarose gel, and the remaining PCR product was purified using the QIAquick PCR Purification Kit (Qiagen, Hilden, Netherlands), following the manufacturer’s protocol.

### Library preparation

Hereafter, library preparation is described for samples that had no Illumina (Illumina, San Diego, CA, USA) adapter attached to the 4C primer. Samples of each replicate were pooled in equimolar amounts, and assessed on a Bioanalyzer (Agilent Technologies, Santa Clara, CA USA). Finally, each sample volume was adjusted to 100 μl using H_2_O. Replicates were then split into two aliquots of 50 μl each, and 10 μl of Resuspension Buffer (RSB; Illumina) and 40 μl End-Repair Mix (ERP) (Illumina) was added. The mixture was incubated for 30 minutes at 30°C. Then, 100 μl of Agencourt AMPure beads (Beckman Coulter, Brea, CA, USA) were added, and the mixture was incubated for 15 minutes at room temperature. The reaction tubes were then placed on a magnetic stand. The supernatants were removed without disturbing the beads, and 400 μl of freshly prepared 80% ethanol was added. After 30 seconds, the ethanol was replaced with another 400 μl of 80% ethanol. The supernatant was removed, and the tubes were left open to dry. The beads binding the 4C PCR products were resuspended in 17.5 μl RSB, and incubated for 2 minutes before being placed on a magnetic stand for 15 minutes. Finally, 15 μl of sample was transferred to a fresh 0.2 ml reaction tube. To each sample, 2.5 μl of RSB and 12.5 μl A-tailing Mix (ATL) (Illumina) were added and mixed thoroughly, followed by incubation at 37°C for 30 minutes. Following this, 2.5 μl of RSB, 2.5 μl of DNA Ligase Mix (LIG) (Illumina) and 2.5 μl of indexed DNA adapters (Illumina) were added, and mixed gently by pipetting the mixture up and down. Subsequently, the mixture was incubated for 10 minutes at 30°C. To inactivate the reaction 5 μl of Stop Ligase Mix (STL) (Illumina) were added, and samples were transferred to a fresh 1.5 ml reaction tube. Then 42.5 μl of Agencourt AMPure beads (Beckman Coulter) were added to each tube, and the mixture was incubated for 15 minutes at room temperature. The tubes were subsequently placed on a magnetic stand for 2 minutes, then 80 μl of supernatant were removed and replaced with 200 μl of freshly prepared 80% ethanol. After incubation for 30 seconds, the supernatant was removed, and the tubes were left open to dry. The previous ethanol washing step described above was repeated once, then, the pellet was resuspended in 52.5 μl RSB. After 2 minutes of incubation at room temperature, tubes were placed on a magnetic stand for 2 minutes, then 50 μl of the supernatant were transferred to a fresh 1.5 ml reaction tube. The Agencourt AMPure (Beckman Coulter) cleanup was repeated once; however, at the final step, instead of being suspended in 52.5 μl RSB, the pellet was resuspended in 22.5 μl RSB, of which 20 μl were transferred to a fresh 0.2 ml reaction tube. Samples with adapters already attached to the 4C PCR primers were treated in the same way from this point on. To perform final library amplification, 5 μl of PCR Primer Cocktail (PPC) and 25 μl of PCR Master Mix (PMM) (both Illumina) were added to each tube. PCR was performed under the following conditions: 98°C for 30 seconds; then 12 cycles of 98°C for 10 seconds, 60°C for 30 seconds, and 72°C for 30 seconds; followed by a final elongation at 72°C for 5 minutes. Samples were then transferred to a 1.5 ml reaction tube, and 50 ml of Agencourt AMPure beads (Beckman Coulter) were added. After 15 minutes of incubation at room temperature, the tubes were placed on a magnetic stand for 2 minutes. Following this, 95 μl of supernatant were removed, and the beads were washed twice with 200 μl of freshly prepared 80% ethanol. After the supernatant was removed, tubes were left open to dry. The pellet was then resuspended in 32.5 μl RSB and incubated for 2 minutes at room temperature. The tubes were placed on a magnetic stand, and 30 μl of the purified library were transferred to a fresh 1.5 ml reaction tube. From each library a 10 nmol/l stock in Tris-Cl (pH 8.5) with 0.1% (v/v) Tween 20 was prepared. All replicates in the libraries were subsequently pooled, and used for Illumina HiSeq 100 bp single end sequencing. For each batch of replicates, one lane per replicate was loaded (total of four lanes). Batch 1 replicate A had a total yield of 92,063,669 raw reads, with a mean quality score of 35.35. Batch 1 replicate B had a total yield of 80,777,012 raw reads with a mean quality score of 35.31; batch 2 replicate A had a total yield of 43,296,252 raw reads with a mean quality score of 36.85; and batch 2 replicate B had a total yield of 55,187,969 raw reads with a mean quality score of 36.76.

### 4C sequencing data pre-processing

The two fastq files (one per replicate) were split into separate viewpoints according to the 4C primer sequences and the *Hind*III restriction pattern within the reads. No mismatches were allowed, and the remaining reads were discarded. After removal of primer and restriction site sequences, reads were trimmed to 30 bp and aligned to the *Arabidopsis* reference genome [48] using bowtie (version 0.12.7) [49] with the command line arguments -a -v 0 -m 25. For alignment statistics, see Additional file 17: Table S2.

Reads with multiple alignments were processed as described previously [50]. Because we estimated the length of a single interaction unit as 100 kb, we used an allocation distance of ±50 kb. To specify potential 4C fragments, we generated an *in silico Hind*III digest of the *Arabidopsis* Col-0 genome. Reads mapping to the ends of the resulting fragments were considered for further analysis. For a more robust measure of interactions, fragments were then used to generate windows spanning a larger region of the genome (that is, 100 fragments, corresponding to 180 kb on average). During this process, fragments closer than 1 kb to the viewpoint were discarded, given that a large proportion of their reads would probably originate from incomplete digestion and/or self-circularization. Furthermore, we discarded all fragments closer than 100 kb to a centromere, as the quality of alignments to centromeres is low. Finally, fragments whose distance from the primary restriction site to the first occurring secondary restriction site was 1000 bp or more with respect to both ends of the fragment were also removed. As a measure of interaction of a given window (interaction value), fragment counts were log-transformed to avoid high impact of outlier fragments, and then summed. Depending on the downstream analysis, windows spanned either 100 fragments from each fragment on (overlapping) or 25 fragments starting from every 25th fragment (non-overlapping).

Processed 4C data files (split according to primer sequence) and raw-data sequencing files are publically available on Gene Expression Omnibus (GEO), accession number GSE50181.

### Data processing of histone modifications, transcription, DNA methylation, and genomic sequencing

To add additional information, such as histone modification patterns and transcription rates, we obtained publicly available data from GEO [51], specifically ChIP sequencing (ChIP-seq) data GSM701923, GSM701924, GSM701925, GSM701926, GSM701927, GSM701928, GSM701929, GSM701930, GSM701931 [30], and RNA-seq data GSM701934 [30]. Pre-processed DNA methylation data was obtained from [32].

ChIP-seq and RNA sequencing (RNA-seq) reads (SOLiD sequencing, 50 bp (Applied Biosystems/Life Technologies) were aligned to the *Arabidopsis* reference genome (Col-0, TAIR10 [52]) using bowtie (version 0.12.7) with the following command line arguments: –a –v 2 –m 25. Reads with multiple alignments were processed as described previously [50]. Allocation distances were set to ±5 kb and ±50 bp for the ChIP-seq and the RNA-seq data, respectively. Histone modification densities and DNA methylation densities were calculated by the sum of nucleotides covered by at least one uniquely alignable short sequence, divided by the total number of nucleotides for each individual 4C restriction fragment.

To estimate potential biases related to sequence composition (such as repetitive sequences), we obtained genomic DNA sequencing data (Illumina, 100 bp) of the data set GSM567816, and processed them identically to the 4C sequencing data.

### Assigning *P*-values to individual windows

To estimate the significance of an interaction, we calculated for each window the probability (that is, *P*-value) to observe its interaction value by chance. Given that an interaction of two fragments would lead to a higher read count in the neighboring fragments as well (hence in the window), random shuffling of fragment positions and recalculation of window interaction values provides randomized interaction data with the values following a normal distribution. Using the parameters of this distribution, a preliminary *P*-value was then calculated for each window. We repeated this process 1,000 times, and averaged for each window the *P*-values from all individual repetitions to obtain a final *P*-value. To take into account the differences between chromosome arms (for example, the different amount of DNA between the short arm and the long arm of chromosome 2), the *P*-values were calculated for each chromosome arm separately.

*P*-value thresholds were chosen to best fulfill the requirements of either plotting or data analysis. Generally, we set the threshold for prey regions to 10^-3^. In the Circos plot of Figure 5A we chose *P* ≤ 10^-4^ for better visibility. Because for various viewpoints, a threshold of 10^-3^ did not yield a sufficient number of prey regions for robust data analysis, we chose a threshold of *P* ≤ 0.05 to perform PCA.

### Distance decay

We estimated the genomic distance-dependent decay of the interaction probability on a distance of 1 kb to 10 Mb from the viewpoint. This stretch was log-transformed, and split into 41 intervals with length of 0.1 (on the log scale). For each sample, the reads of the fragments corresponding to the intervals were summed up and assigned to the interval. Given that the centromere acts as an interaction boundary, only fragments on the viewpoint's arm were considered. Read counts per interval were then divided by the total number of reads across all intervals representing contact probabilities, which across the full distance add up to 1. Given that some intervals contained only a few fragments and, in certain cases, only fragments from a subset of the viewpoints, we used a locally weighted scatterplot smoothing (LOESS) predictor fitted to the original data to calculate one single contact probability value for each interval. To obtain the slope, and hence the distance decay coefficient, we then approximated the data with a linear model. Slope and *P*-value were derived from the fit of the linear model to the values predicted by the LOESS fit. However, direct fitting of a linear model to the original data yielded almost equal results with a slope of −0.72 instead of −0.73, and an extremely low *P* value (<10^-100^).

### Centromere distance

To analyze the effect of a viewpoint's distance to the centromere on the distribution of the observed interaction frequencies along chromosome arms, we calculated for each chromosome arm (except the viewpoint's arm) the distance to the centromere at which 50% of all reads were aligned, and then fitted a linear model. The procedure was performed twice, first using absolute values, and then relative distances, defined as the absolute distance divided by the length of the chromosome arm (transformed by taking the arcsine of the square root).

### Principal component analysis

All PCAs were based on non-overlapping windows that included 25 fragments. For each viewpoint, mean prey and control histone densities for each histone modification (that is, EMD) were calculated. Subsequently, PCA was performed on a dataset including mean EMD values of control and prey regions for each viewpoint and EMD. PCA was performed using the built-in R princomp() function.

### Permutation test

To analyze differences in the epigenetic landscape of prey and control regions, we randomly selected 50 prey and 50 control regions (sampled) for each viewpoint, and obtained a corresponding randomized test set by pooling their EMDs and permuting them (shuffling them into two randomized groups of 50 values each). We then calculated the absolute differences in averaged EMDs between the sampled (RealDiff_ij_), and the permutated (RandDiff_ij_) prey and control regions, respectively.

Repeating this step *i* times for each of the *j* viewpoints yielded an empirical distribution for RandDiff for every epigenetic modification with 13,000 values (j = 13 viewpoints, and i = 1,000 repetitions). Comparing the average RealDiff_m_ (mean across all repetitions and viewpoints) with this distribution then provided an empirical *P*-value (p = ∑(RandDiff_ij_ > RealDiff_m_)/(i*j)), which was subsequently adjusted for multiple testing calculating false discovery rate (FDR; Benjamini-Hochberg).

### Analysis of individual epigenetic marks employing GSEA-like analysis

To test whether prey regions have a different epigenetic landscape from that of regions chosen randomly across the genome, we developed a procedure similar to the GSEA described previously [33]. It requires densities of EMDs (for example, CG methylation density or H3K9me2) assigned to all (*n*) regions in the genome (that is, non-overlapping windows spanning 25 restriction fragments), and a subset (*m*) of the regions as a test set (that is, prey regions with a *P* < 0.01 in both replicates). During the procedure, the regions are first sorted according to their EMD. We then assigned a value of −1 to regions not in the test set, and a value of (*n-m*)*/m* to the regions in the test set (to assure that the sum of these values across all regions would be zero). In a third step, the cumulative sum of these values was calculated and the enrichment score (ES) was defined as the maximum (absolute) deviation from zero. If the regions in the test set were randomly distributed across the sorted list of all regions, the cumulative sum would fluctuate around zero with a relatively small ES. Conversely, a non-random distribution of the test set (for example, accumulation at one end of the sorted list) would lead to a high ES. A *P*-value could then be assigned by comparing an observed ES to an ES distribution obtained by randomly choosing *m* regions 10,000 times. To obtain one *P*-value per epigenetic feature, the ES were averaged across all viewpoints. As we were focusing on long-range interactions, we excluded all interactions within the viewpoint’s arm. Because statistical testing for all epigenetic features was employed, using the same 4C data, *P*-values were adjusted for multiple testing, calculating FDR (Benjamini-Hochberg).

### Plotting

All plotting of 4C data, genomic features, and histone modification data was performed using either Circos [23] or built-in R functions [53] plotting. Code is available upon request.

### Data availability

All sequencing data and processed 4C files are available on Gene Expression Omnibus (GEO) accession number GSE50181.

## Abbreviations

3C: Chromosome conformation capture; 4C: Circular chromosome conformation capture; ChIP-seq: Chromatin immunoprecipitation sequencing; EMD: Epigenetic modification density; ES: Enrichment score; FDR: False discovery rate; FISH: Fluorescent *in situ* hybridization; GEO: Gene Expression Omnibus; GSEA: Gene Set Enrichment Analysis; H3K27me1: Monomethylation of lysine 27 of H3; H3K36me3: Trimethylation of lysine 36 of H3; H3K4me2: Dimethylation of lysine 4 of H3; H3K9me2: Dimethylation of lysine 9 of H3; PCA: Principal component analysis; RNA-seq: RNA sequencing; RPKM: Reads per kilobase per million; RPM: Reads per million.

## Competing interests

The authors declare that they have no competing interests.

## Authors’ contributions

SG conceived the study, conducted the experiments, performed data analysis, and wrote the manuscript. MWS performed data analysis and helped to write the manuscript. TW helped to conceive the study and helped to edit the manuscript. NL helped to conceive the study. UG conceived the study, and helped with data interpretation and writing of the manuscript. All authors read and approved the final manuscript.

## Supplementary Material

Additional file 1: Figure S1 Circular chromosome conformation capture (4C) interactome of *MEA* F6. Click here for file

Additional file 2: Figure S2 Circular chromosome conformation capture (4C) interactome of *MEA* F8. Click here for file

Additional file 3: Figure S3 Circular chromosome conformation capture (4C) interactome of *AT1G51860*. Click here for file

Additional file 4: Figure S4 Circular chromosome conformation capture (4C) interactome of *PHE1*. Click here for file

Additional file 5: Figure S5 Circular chromosome conformation capture (4C) interactome of *FIS2*. Click here for file

Additional file 6: Figure S6 Circular chromosome conformation capture (4C) interactome of *CKI1.* Click here for file

Additional file 7: Figure S7 Circular chromosome conformation capture (4C) interactome of *AT3G44380.* Click here for file

Additional file 8: Figure S8 Circular chromosome conformation capture (4C) interactome of *SWN.* Click here for file

Additional file 9: Figure S9 Circular chromosome conformation capture (4C) interactome of *hk4s*. Click here for file

Additional file 10: Figure S10 Circular chromosome conformation capture (4C) interactome of *YAO*. Click here for file

Additional file 11: Figure S11 Circular chromosome conformation capture (4C) interactome of *AG*. Click here for file

Additional file 12: Figure S12 Circular chromosome conformation capture (4C) interactome of *FWA*. Click here for file

Additional file 13: Figure S13 Circular chromosome conformation capture (4C) interactome of *FLC*. Click here for file

Additional file 14: Figure S15 Principal component analysis (PCA) for individual viewpoints. Each graph represents a bi-plot of a PCA, including histone modification densities (EMDs) for prey and control regions of a given viewpoint, respectively. Contributions to the variance of the first two principal components are indicated below the bi-plot. Loadings of the four major factors to the first principal component are listed. Click here for file

Additional file 15: Figure S16 Epigenetic modification density (EMD). For each EMD and viewpoint, the mean EMD for 1,000 × randomly chosen 50 prey and control regions was calculated and plotted. Green bars, prey; yellow bars, control. Click here for file

Additional file 16: Figure S14 Interaction frequency decay for individual viewpoints. Interaction frequency decay is plotted for individual viewpoints. Black line: LOESS smoothened decay. Red dotted line: Linear regression. Values of the slopes are indicated in the lower left corner of each graph. Click here for file

Additional file 17: Table S1 Viewpoint coordinates and primer sequences. Indicated are the viewpoints’ names, their respective chromosome and position in bp, primer sequences, and restriction enzymes used for primary (1°RS) and secondary (2°RS) digest, respectively. **Table S2.** Alignment scores. Columns indicating chromosomes show numbers of mapped reads. Other columns show unmapped reads, percentage of mapped reads, and total reads. Click here for file
