# Supplementary figures and images for: Characterization of chromosomal architecture in Arabidopsis by chromosome conformation capture
Source: Genome Biol. 2013 Nov 24;14(11):R129. doi: 10.1186/gb-2013-14-11-r129 (PMC4053840; doi:10.1186/gb-2013-14-11-r129)

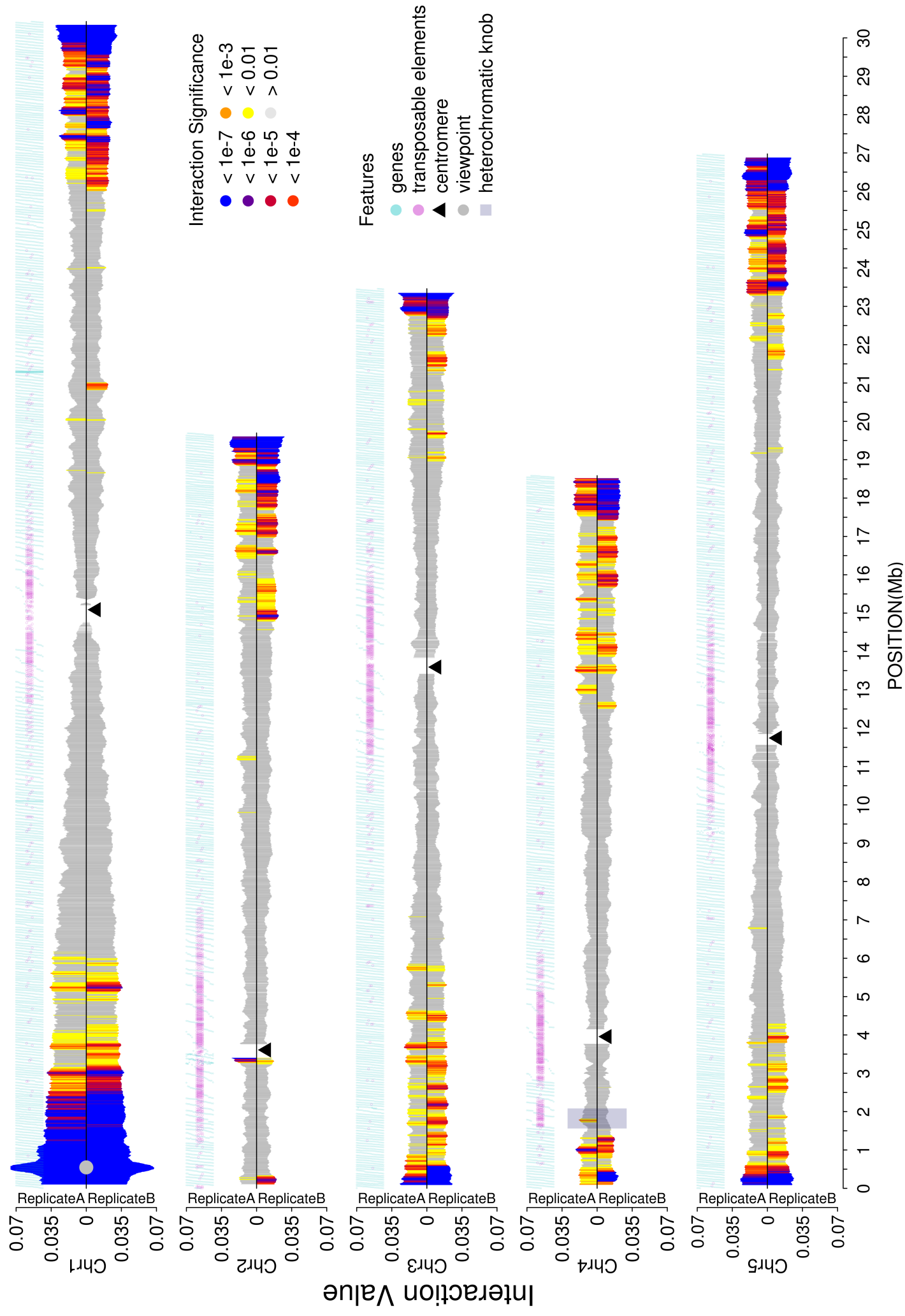

Supplement: Additional file 1: Figure S1 — Circular chromosome conformation capture (4C) interactome of MEA F6. [file gb-2013-14-11-r129-S1.pdf]

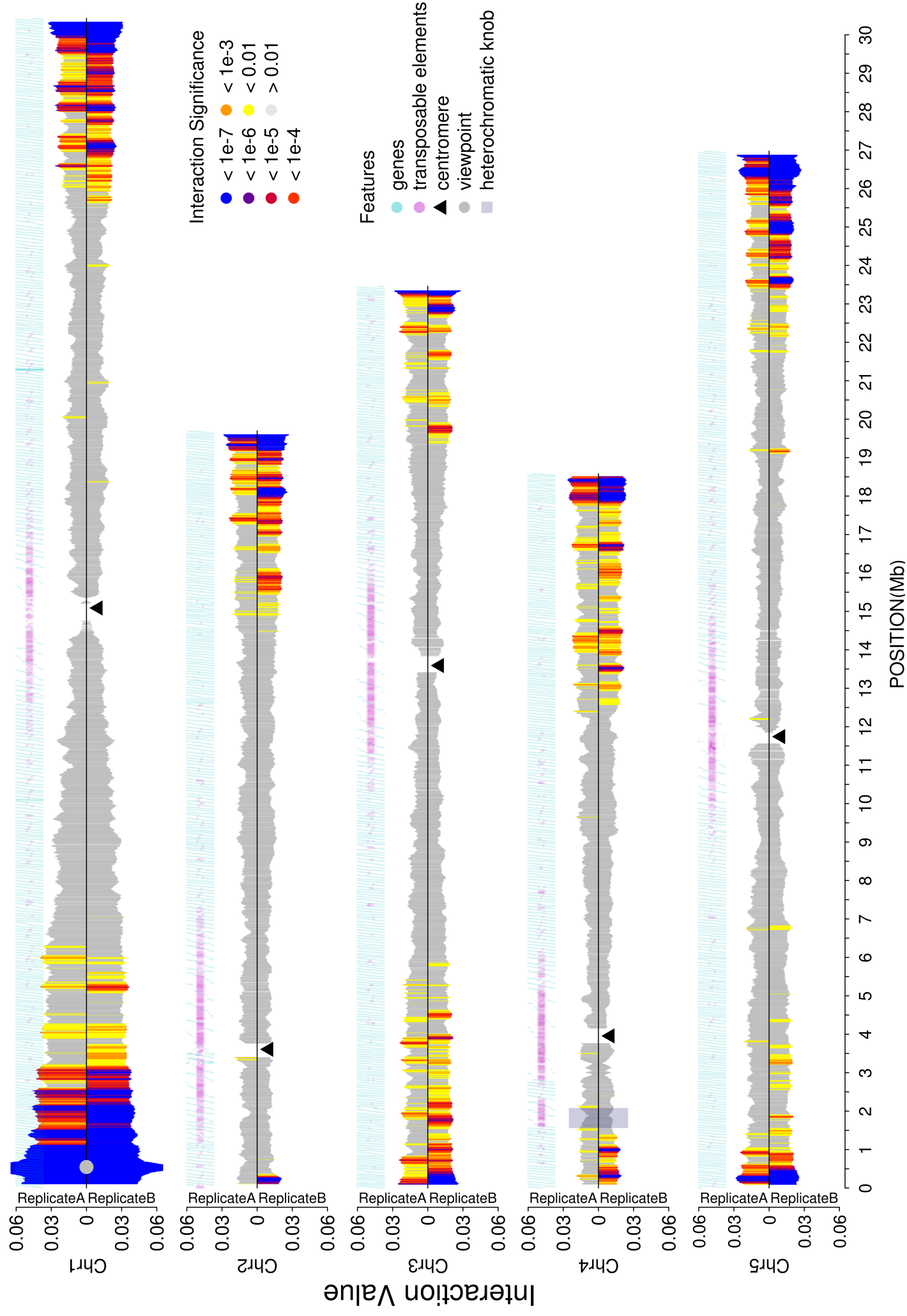

Supplement: Additional file 2: Figure S2 — Circular chromosome conformation capture (4C) interactome of MEA F8. [file gb-2013-14-11-r129-S2.pdf]

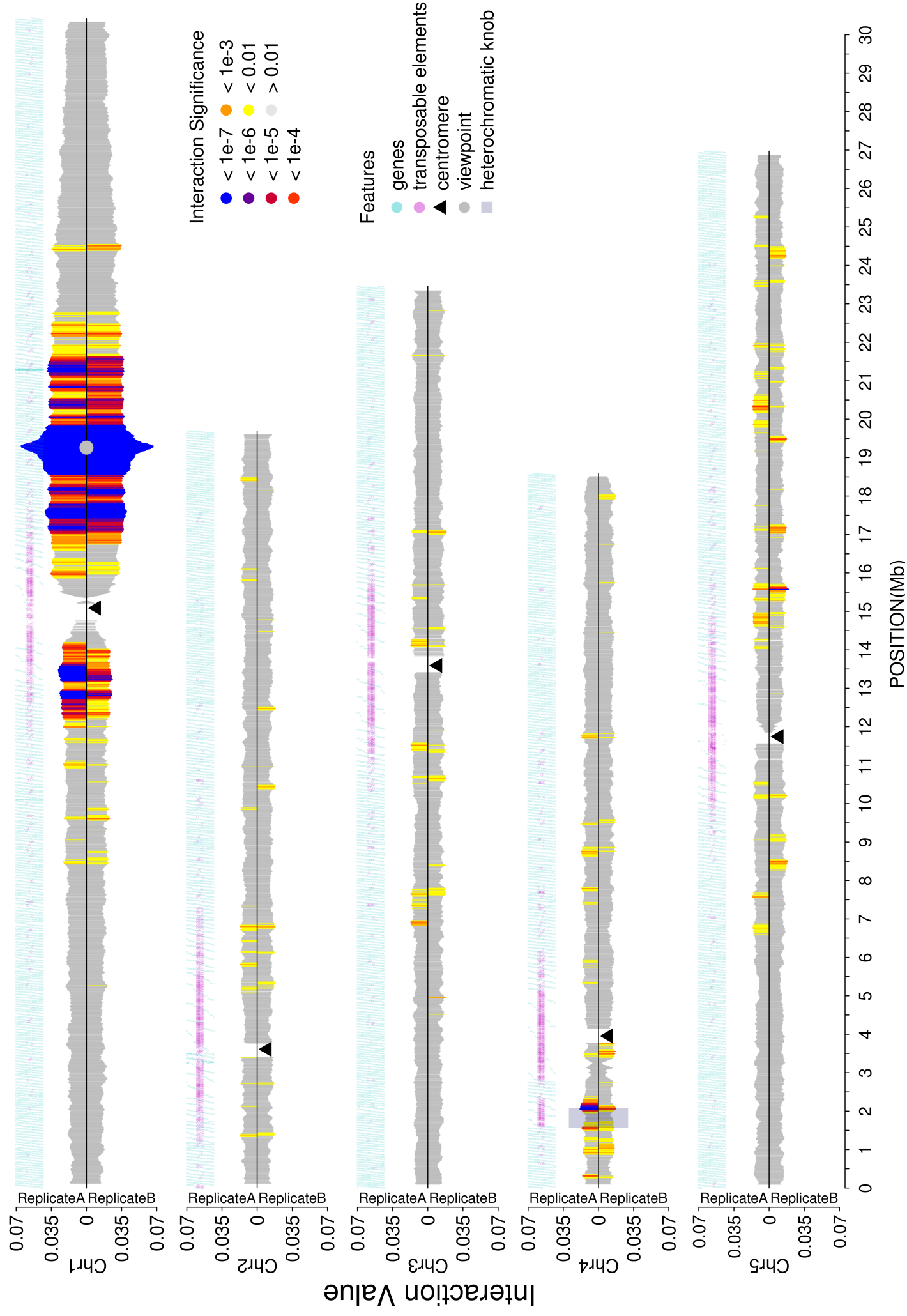

Supplement: Additional file 3: Figure S3 — Circular chromosome conformation capture (4C) interactome of AT1G51860. [file gb-2013-14-11-r129-S3.pdf]

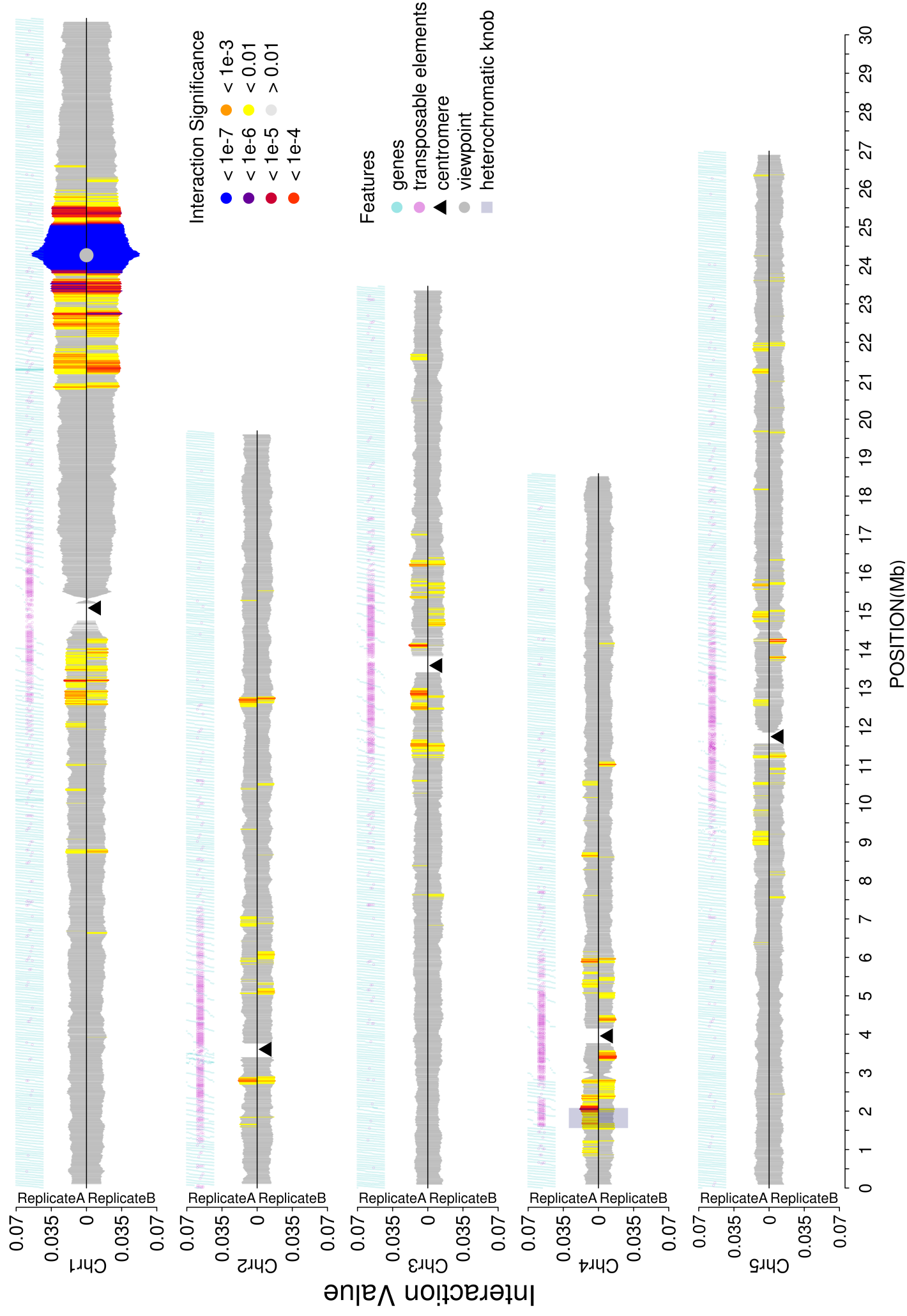

Supplement: Additional file 4: Figure S4 — Circular chromosome conformation capture (4C) interactome of PHE1. [file gb-2013-14-11-r129-S4.pdf]

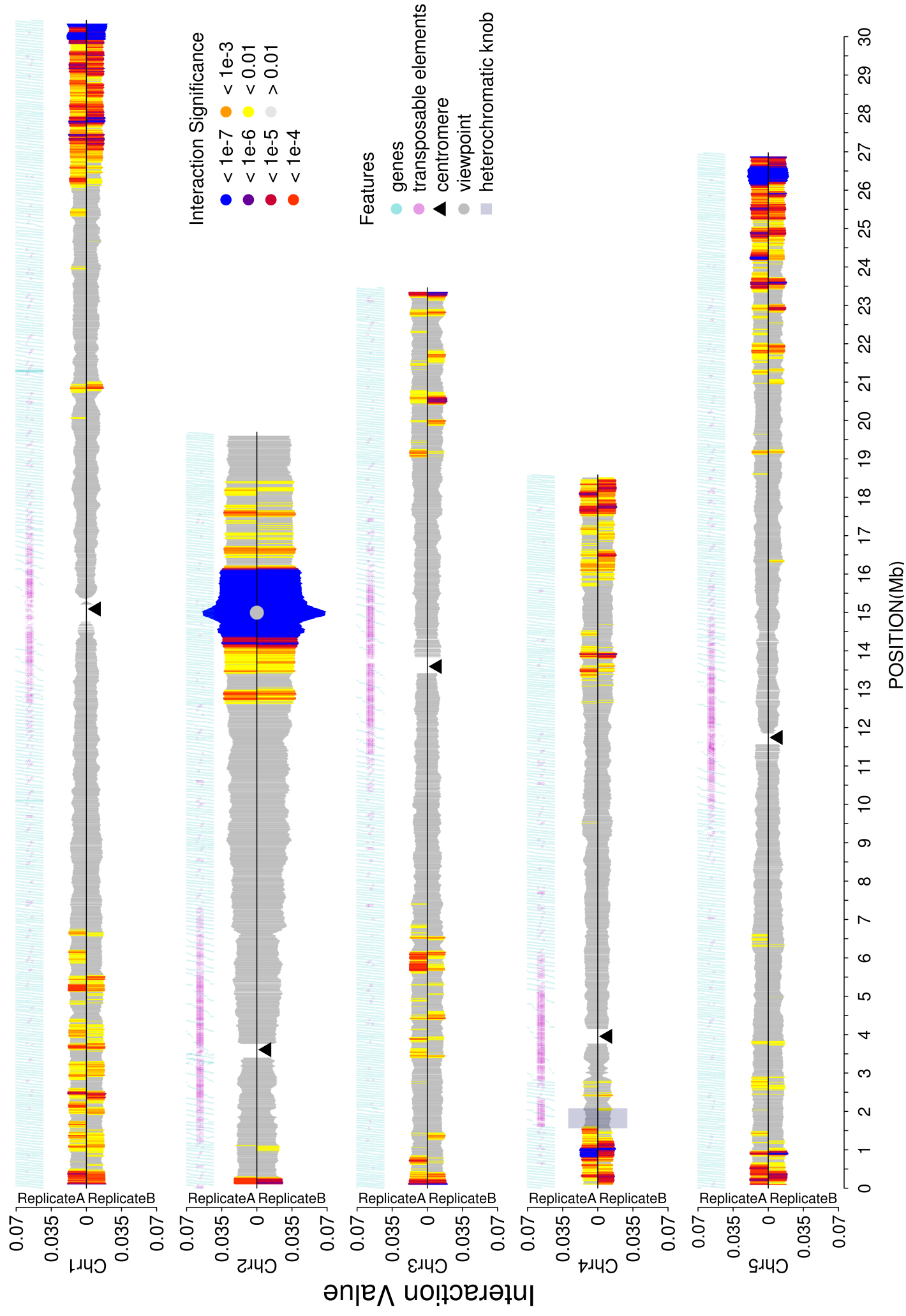

Supplement: Additional file 5: Figure S5 — Circular chromosome conformation capture (4C) interactome of FIS2. [file gb-2013-14-11-r129-S5.pdf]

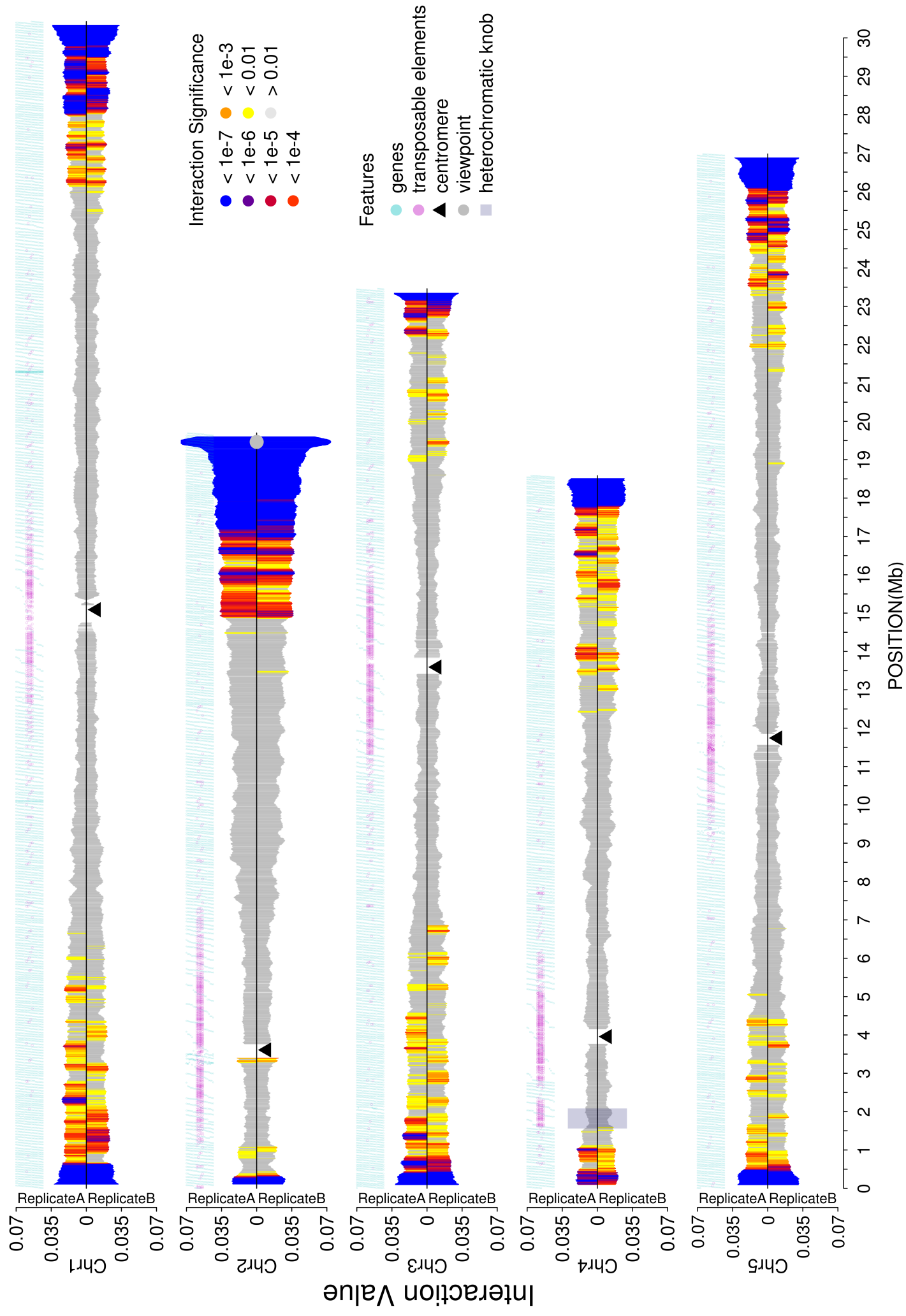

Supplement: Additional file 6: Figure S6 — Circular chromosome conformation capture (4C) interactome of CKI1. [file gb-2013-14-11-r129-S6.pdf]

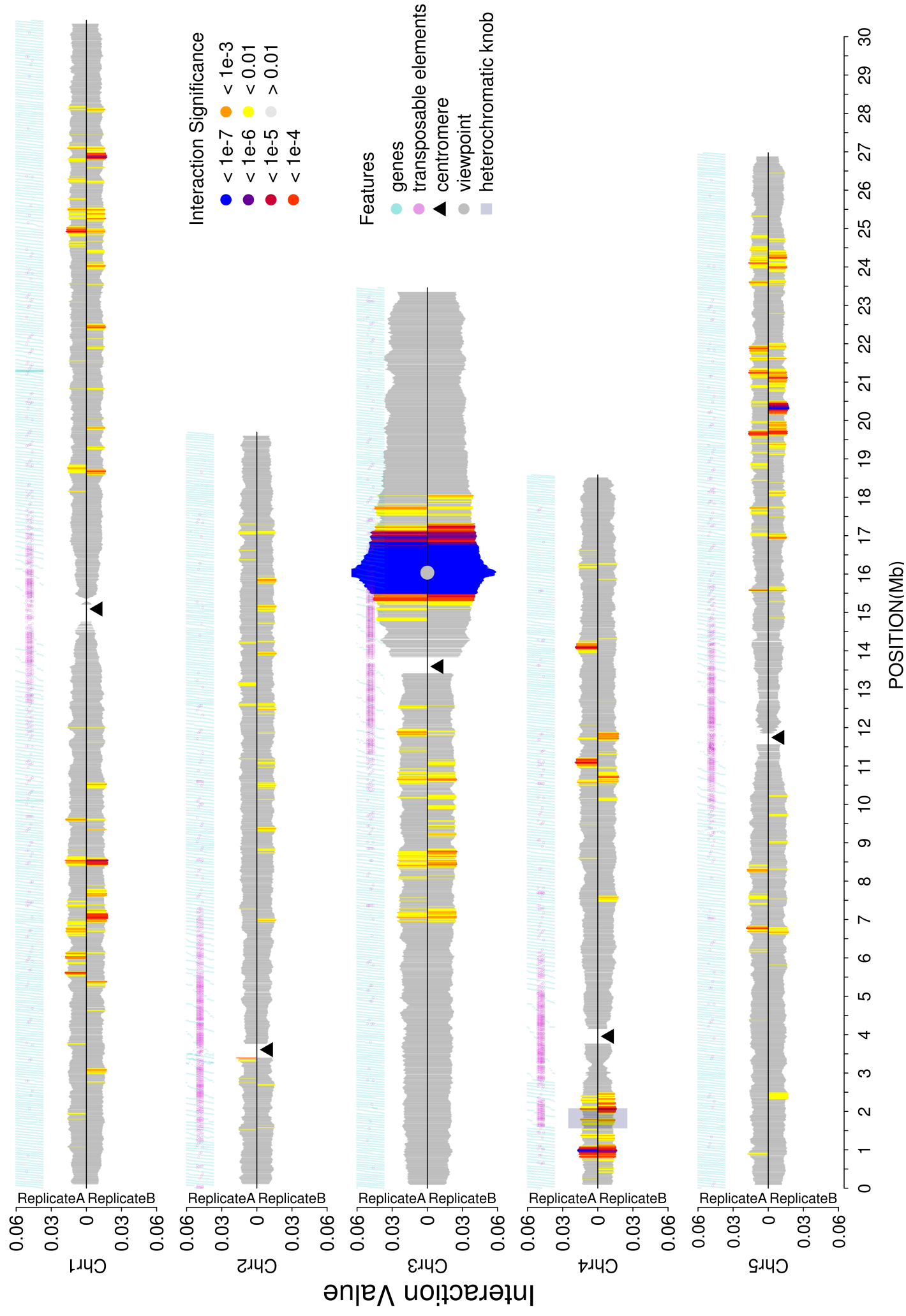

Supplement: Additional file 7: Figure S7 — Circular chromosome conformation capture (4C) interactome of AT3G44380. [file gb-2013-14-11-r129-S7.pdf]

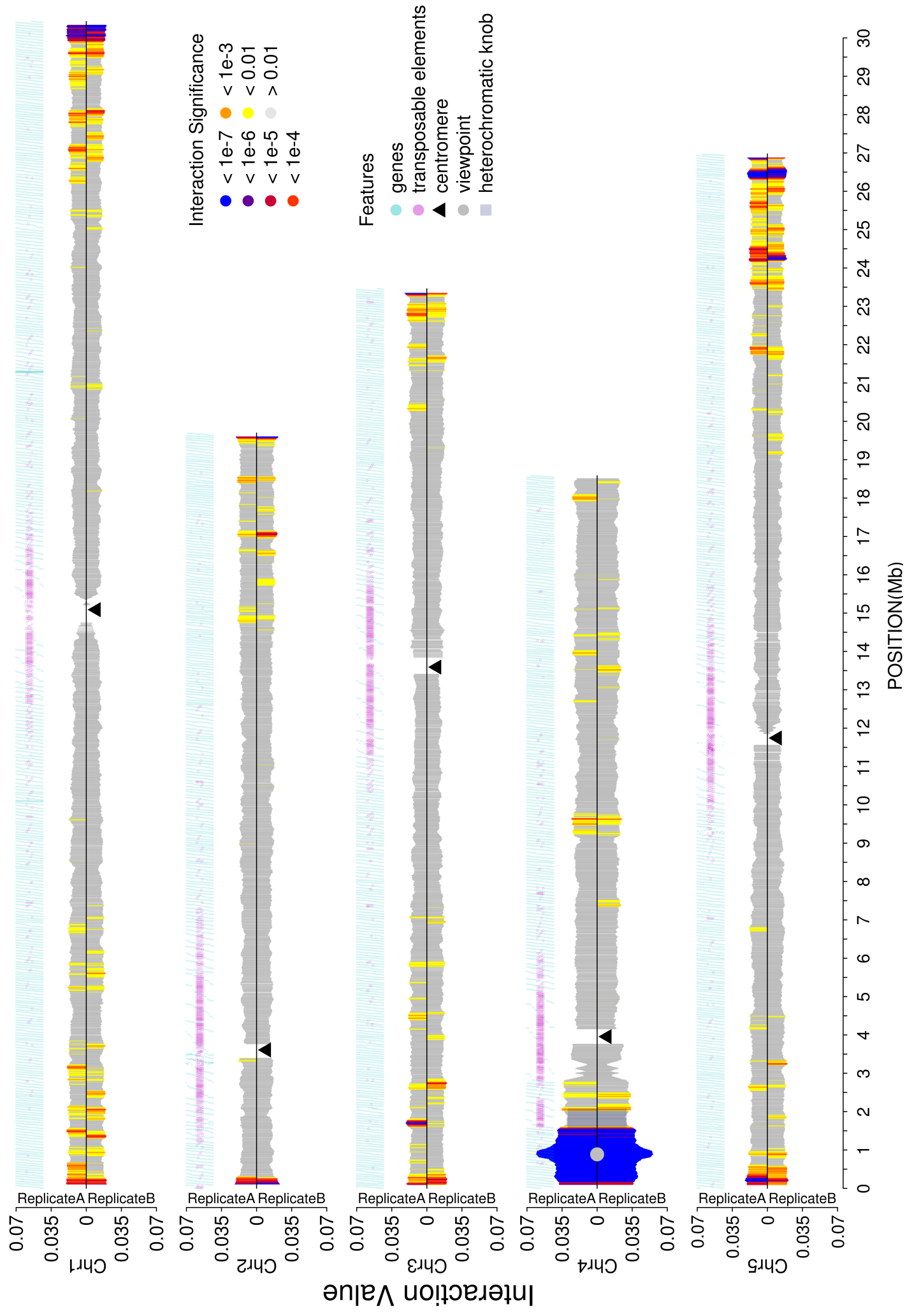

Supplement: Additional file 8: Figure S8 — Circular chromosome conformation capture (4C) interactome of SWN. [file gb-2013-14-11-r129-S8.pdf]

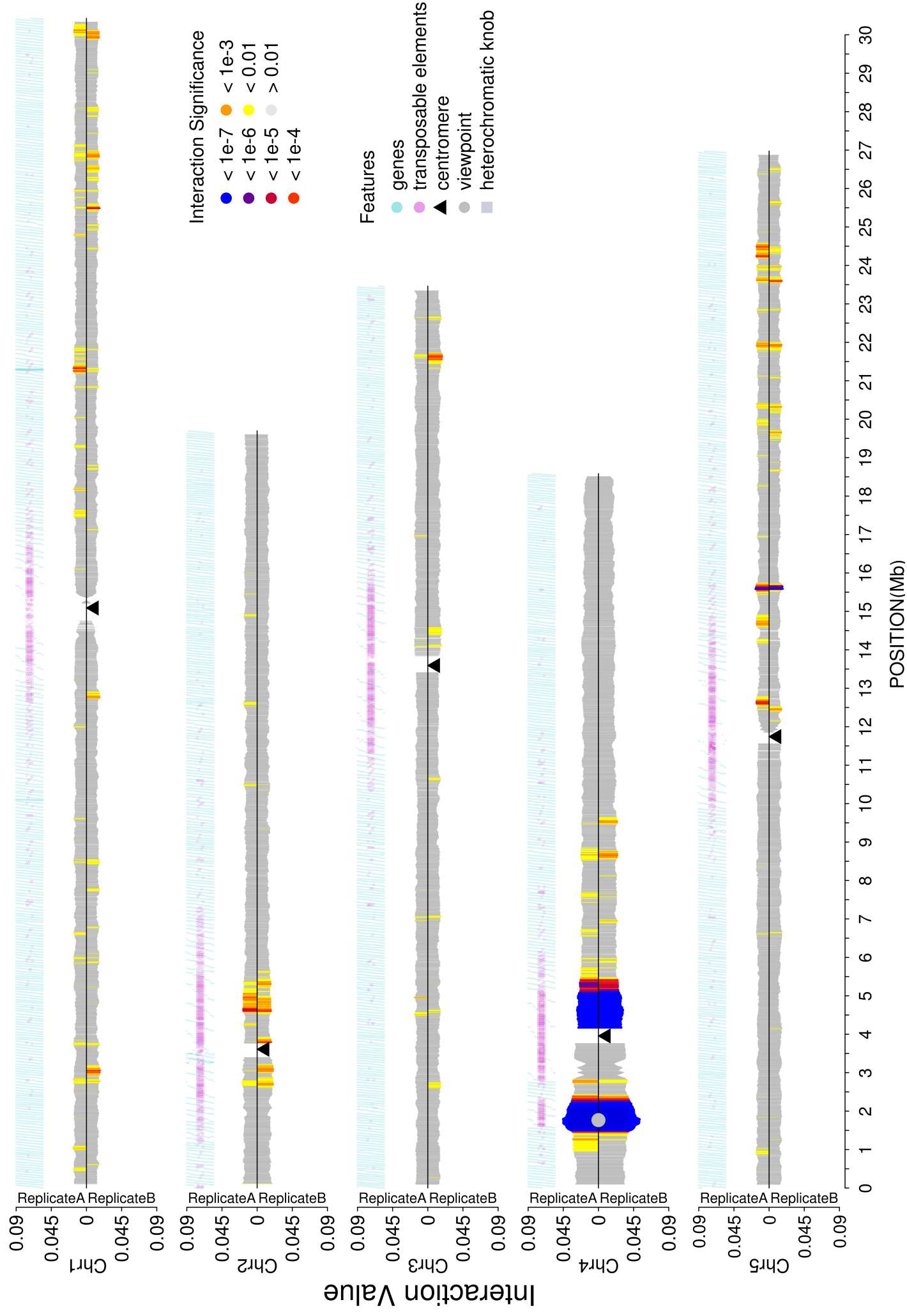

Supplement: Additional file 9: Figure S9 — Circular chromosome conformation capture (4C) interactome of hk4s. [file gb-2013-14-11-r129-S9.pdf]

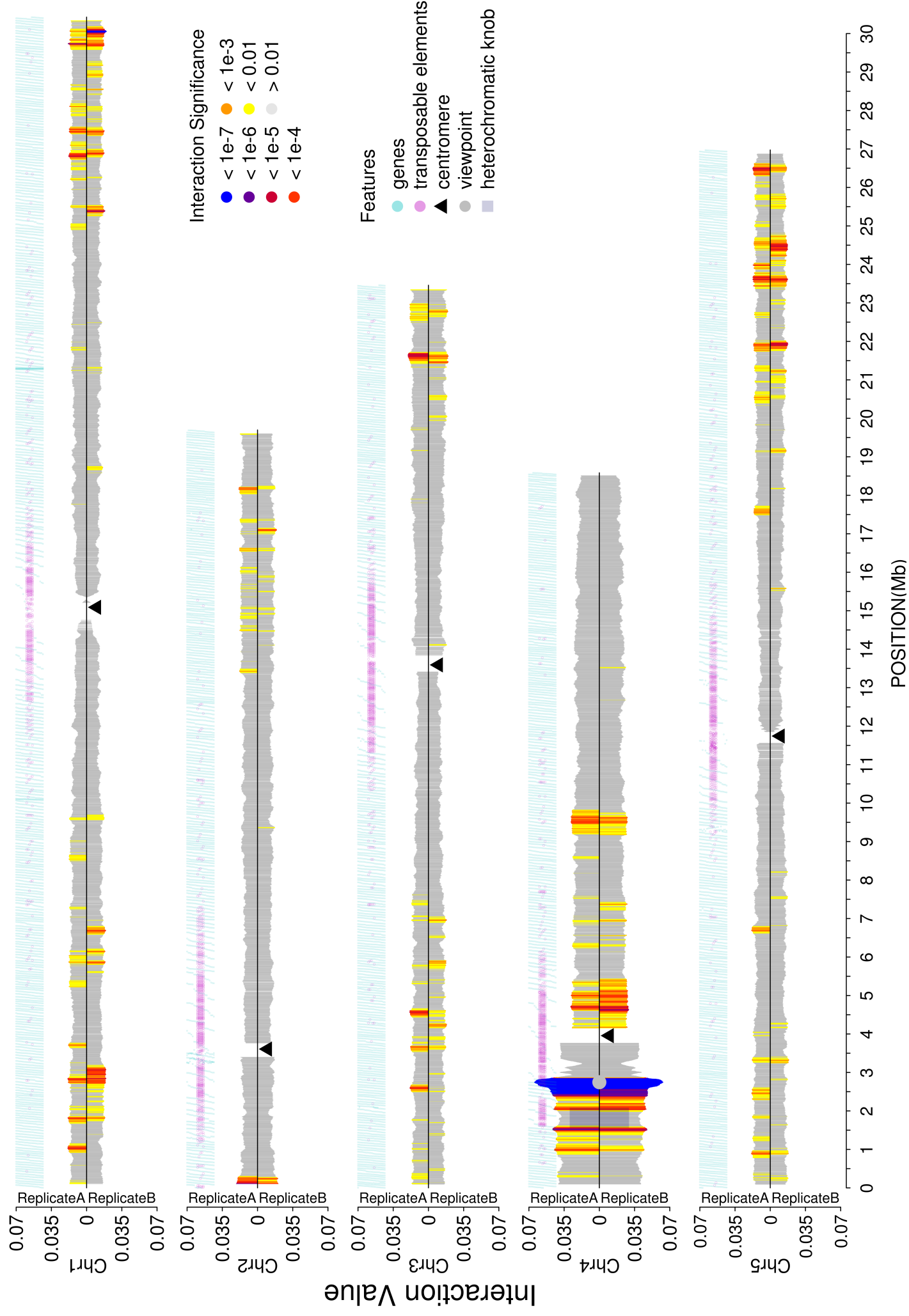

Supplement: Additional file 10: Figure S10 — Circular chromosome conformation capture (4C) interactome of YAO. [file gb-2013-14-11-r129-S10.pdf]

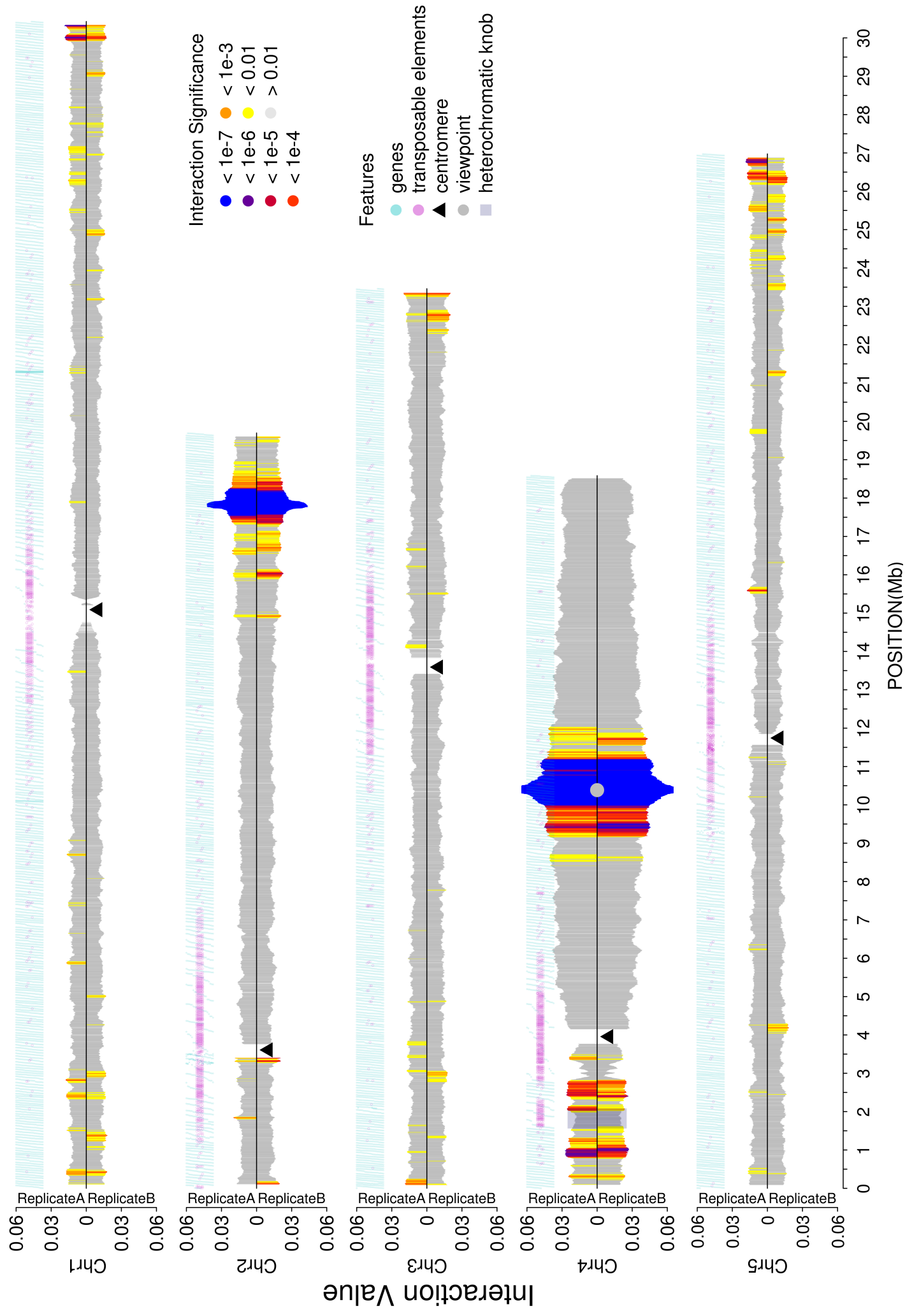

Supplement: Additional file 11: Figure S11 — Circular chromosome conformation capture (4C) interactome of AG. [file gb-2013-14-11-r129-S11.pdf]

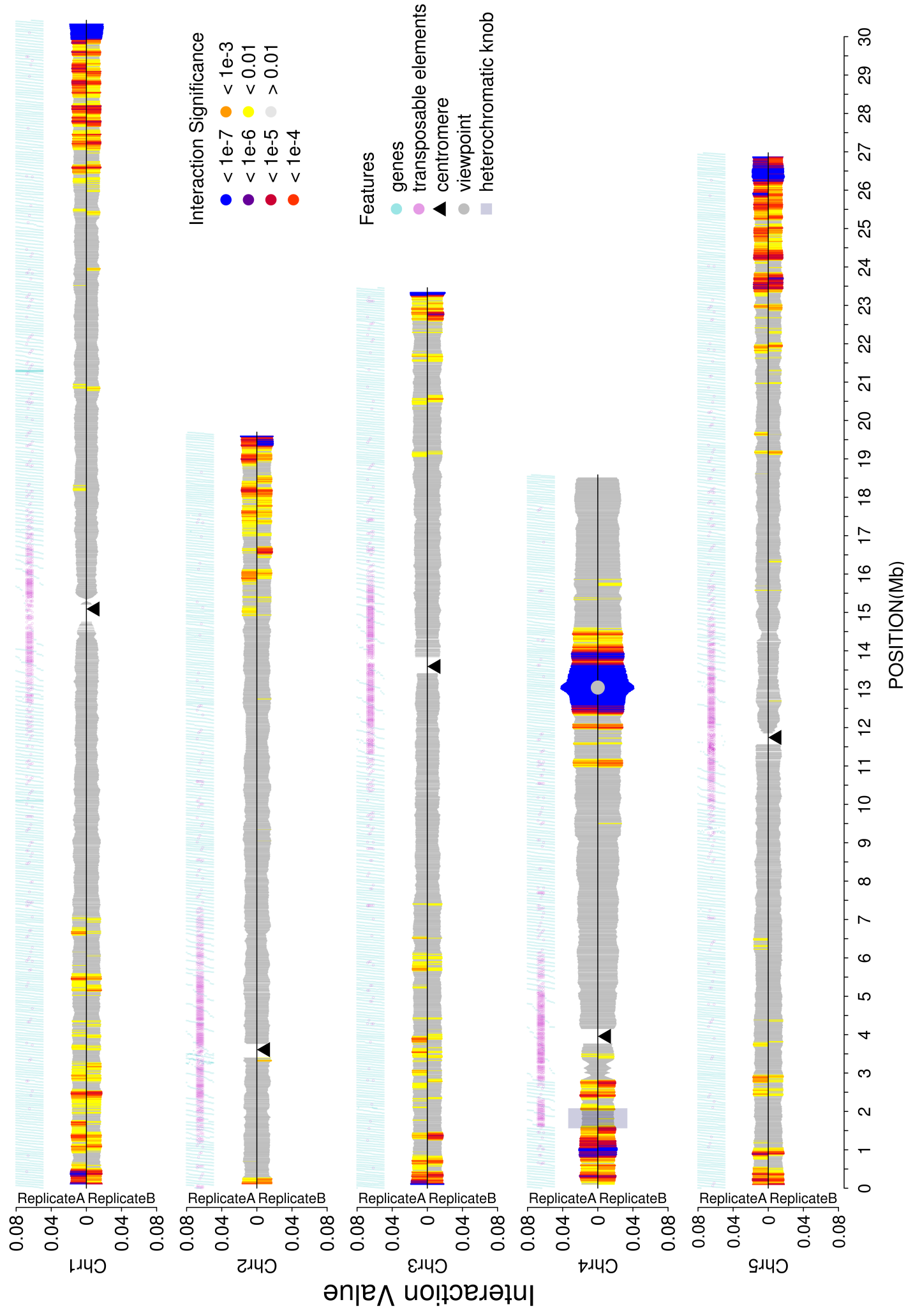

Supplement: Additional file 12: Figure S12 — Circular chromosome conformation capture (4C) interactome of FWA. [file gb-2013-14-11-r129-S12.pdf]

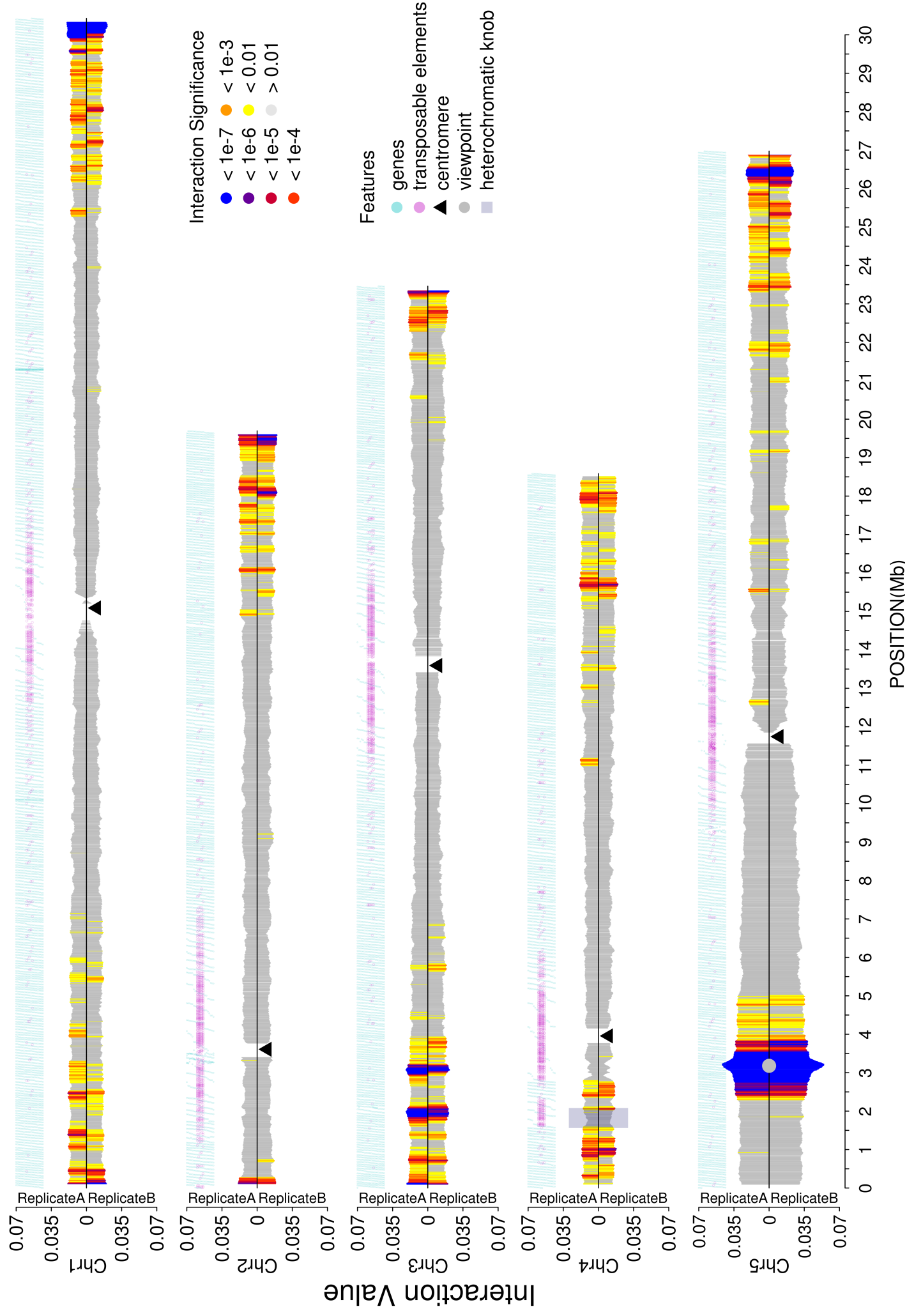

Supplement: Additional file 13: Figure S13 — Circular chromosome conformation capture (4C) interactome of FLC. [file gb-2013-14-11-r129-S13.pdf]

Supplemental Figure 17

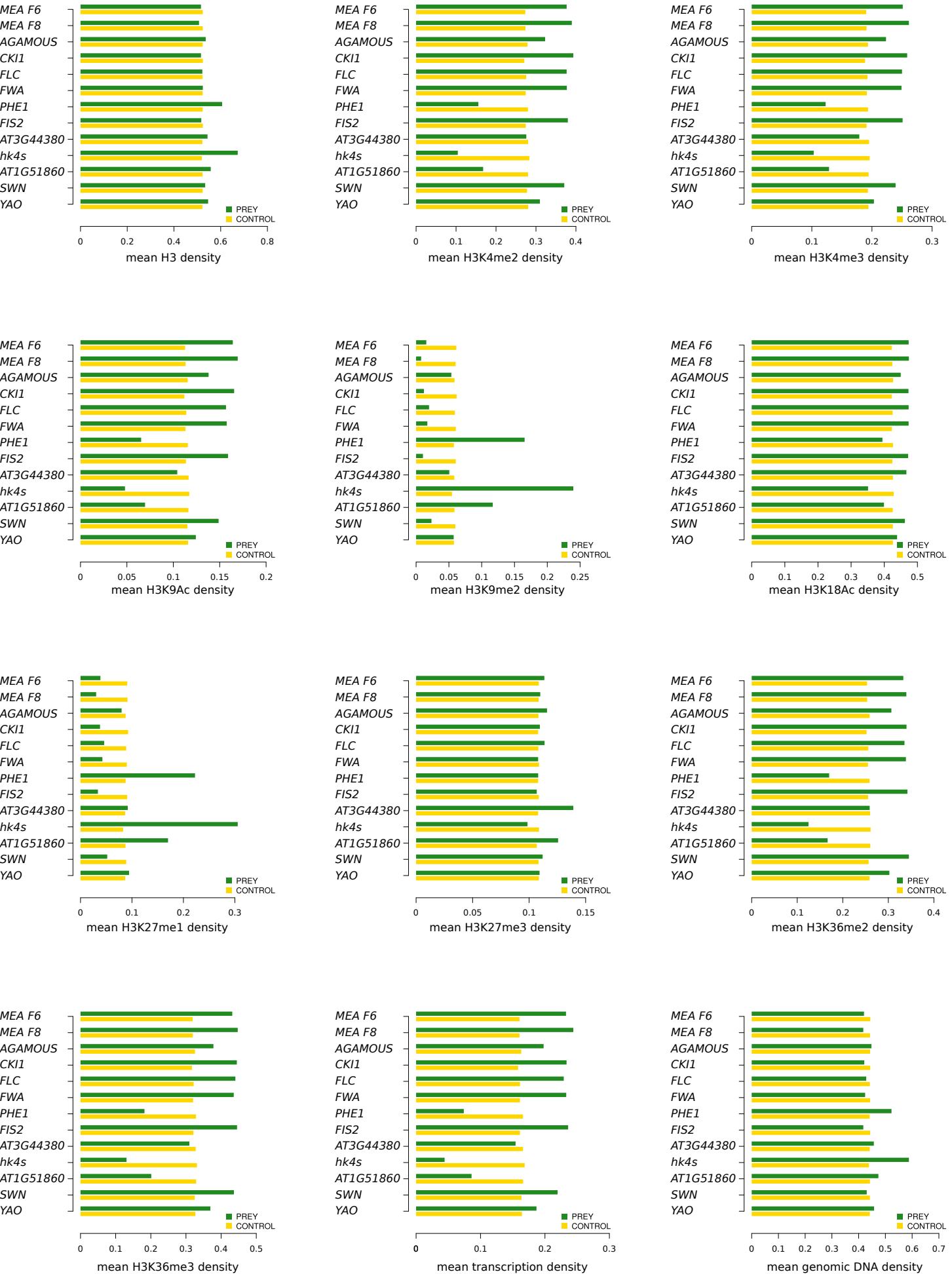

Supplemental Figure 17

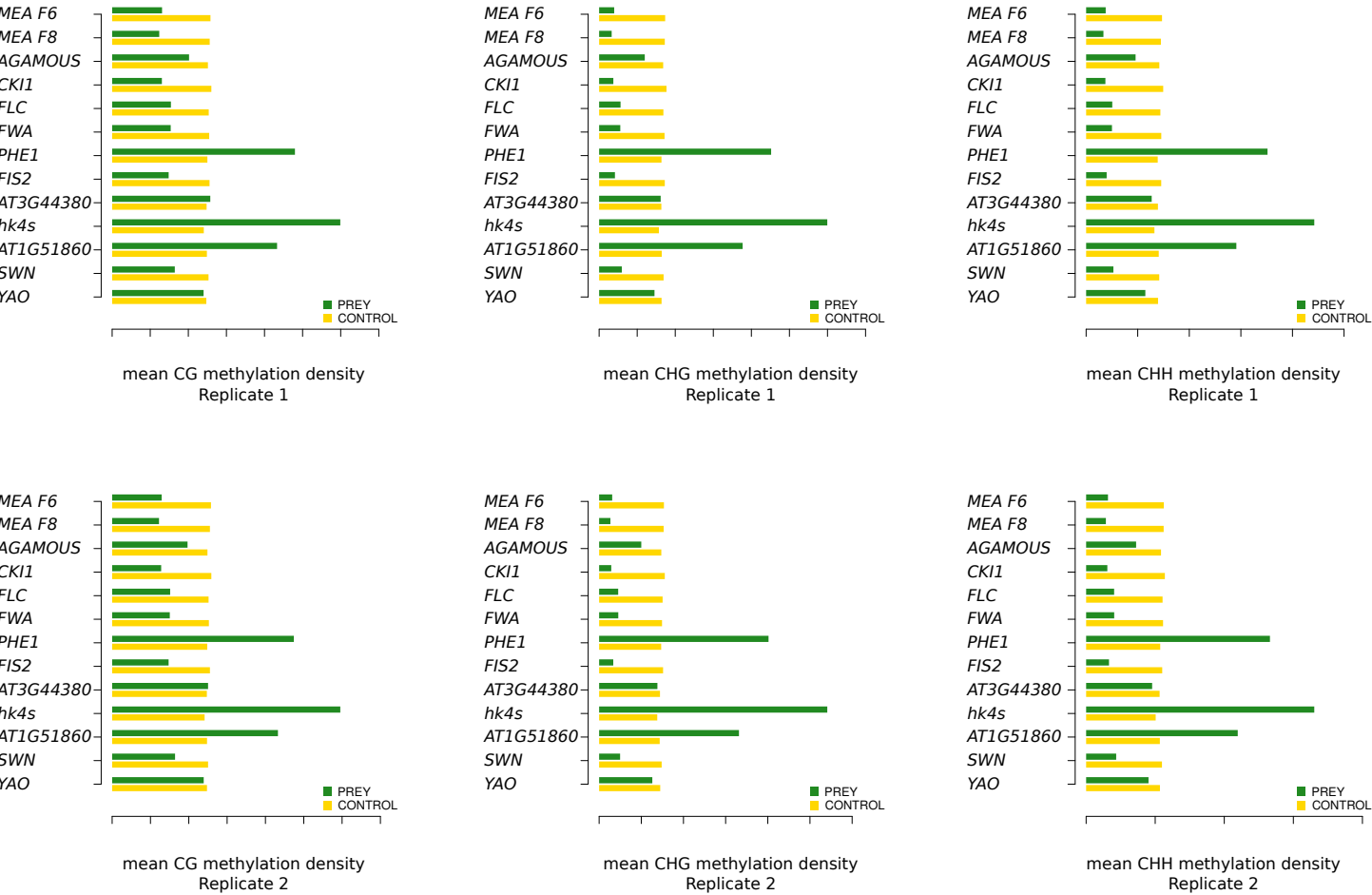

Supplement: Additional file 15: Figure S16 — Epigenetic modification density (EMD). For each EMD and viewpoint, the mean EMD for 1,000 × randomly chosen 50 prey and control regions was calculated and plotted. Green bars, prey; yellow bars, control. [file gb-2013-14-11-r129-S15.pdf]

Supplemental Figure 14

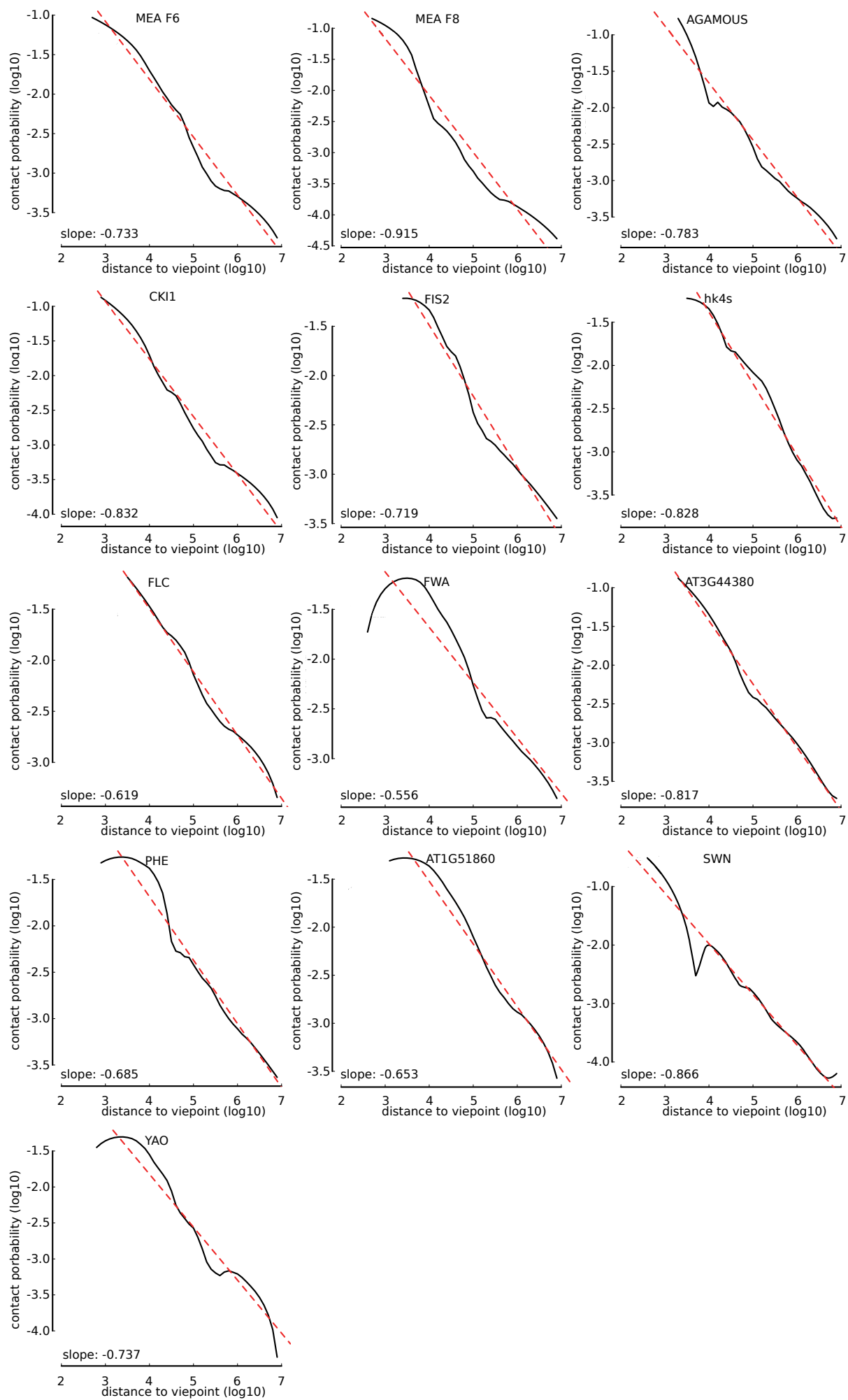

Supplement: Additional file 16: Figure S14 — Interaction frequency decay for individual viewpoints. Interaction frequency decay is plotted for individual viewpoints. Black line: LOESS smoothened decay. Red dotted line: Linear regression. Values of the slopes are indicated in the lower left corner of each graph. [file gb-2013-14-11-r129-S16.pdf]
