# Supplementary material for: Characterization of chromosomal architecture in Arabidopsis by chromosome conformation capture
Source: Genome Biol. 2013 Nov 24;14(11):R129. doi: 10.1186/gb-2013-14-11-r129 (PMC4053840; doi:10.1186/gb-2013-14-11-r129)
Supplement: Additional file 14: Figure S15 — Principal component analysis (PCA) for individual viewpoints. Each graph represents a bi-plot of a PCA, including histone modification densities (EMDs) for prey and control regions of a given viewpoint, respectively. Contributions to the variance of the first two principal components are indicated below the bi-plot. Loadings of the four major factors to the first principal component are listed. [file gb-2013-14-11-r129-S14.pdf]

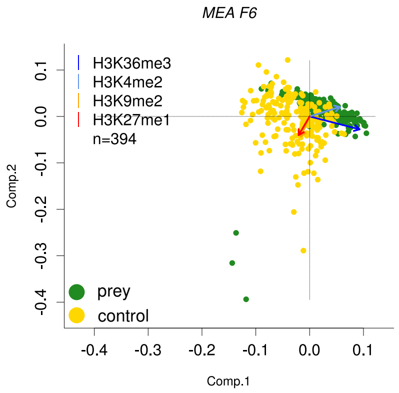

Component 1 explains 36% of total variation
Component 2 explains 14% of total variation

H3K36me3 p = 4.1e-13
H3K4me2 p = 3.5e-22
H3K9me2 p = 0.00024
H3K27me1 p = 5.7e-05

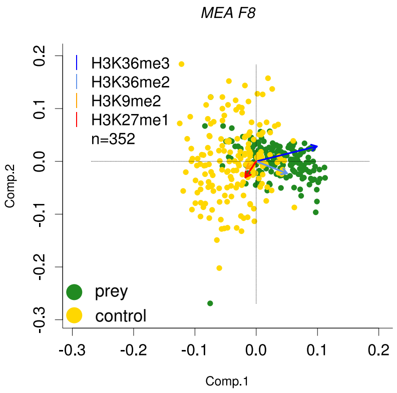

Component 1 explains 36% of total variation
Component 2 explains 12% of total variation

H3K36me3 p = 6.2e-19
H3K36me2 p = 3.5e-16
H3K9me2 p = 2.6e-09
H3K27me1 p = 2.9e-06

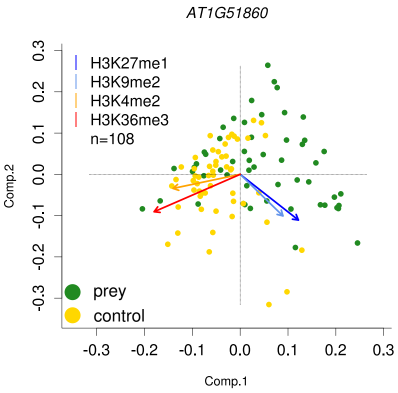

Component 1 explains 39% of total variation
Component 2 explains 18% of total variation

H3K27me1 p = 0.0013
H3K9me2 p = 0.00084
H3K4me2 p = 0.0023
H3K36me3 p = 9.8e-06

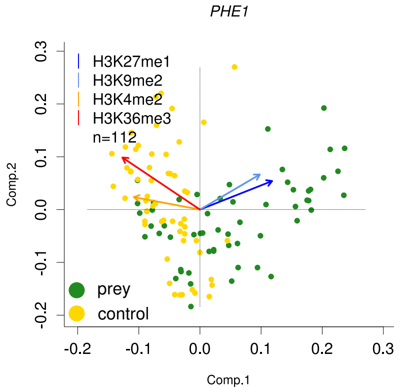

Component 1 explains 49% of total variation
Component 2 explains 14% of total variation

H3K27me1 p = 7.4e-09
H3K9me2 p = 4.5e-07
H3K4me2 p = 1.4e-06
H3K36me3 p = 2.4e-08

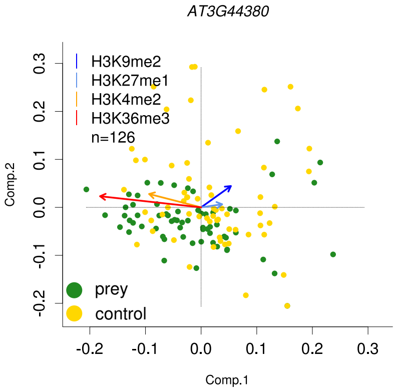

Component 1 explains 33% of total variation
Component 2 explains 14% of total variation

H3K9me2 p = 0.16
H3K27me1 p = 0.27
H3K4me2 p = 0.3
H3K36me3 p = 0.048

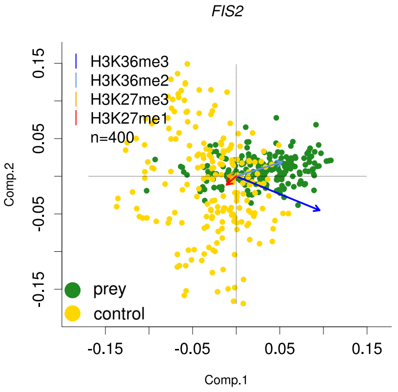

Component 1 explains 34% of total variation
Component 2 explains 13% of total variation

H3K36me3 p = 1e-14
H3K36me2 p = 5.2e-16
H3K27me3 p = 0.54
H3K27me1 p = 5.9e-05

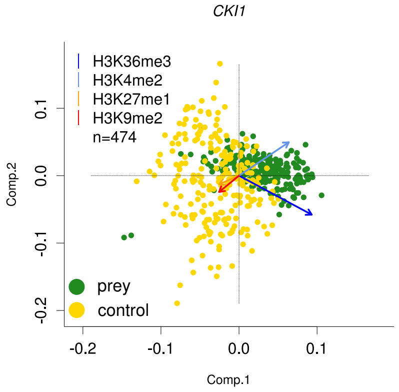

Component 1 explains 35% of total variation
Component 2 explains 14% of total variation

H3K36me3 p = 4.1e-19
H3K4me2 p = 1e-28
H3K27me1 p = 2.5e-06
H3K9me2 p = 3.7e-12

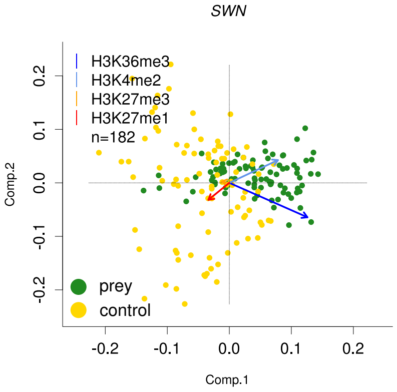

Component 1 explains 37% of total variation
Component 2 explains 14% of total variation

H3K36me3 p = 1.8e-07
H3K4me2 p = 3e-09
H3K27me3 p = 0.17
H3K27me1 p = 4e-04

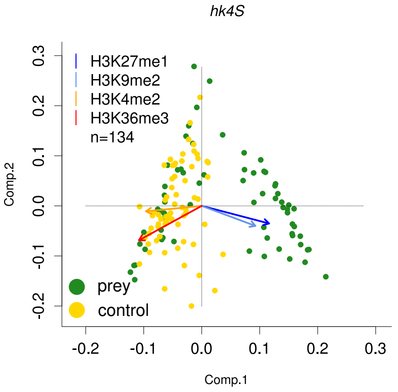

Component 1 explains 59% of total variation
Component 2 explains 12% of total variation

H3K27me1 p = 1.3e-06
H3K9me2 p = 7.2e-05
H3K4me2 p = 3.4e-06
H3K36me3 p = 4.5e-06

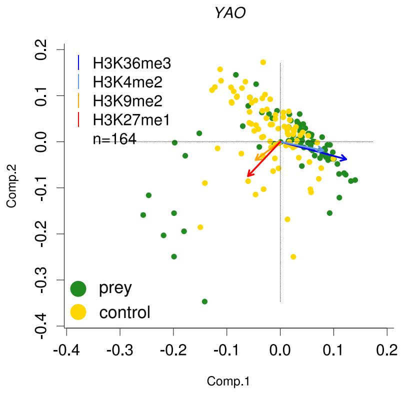

Component 1 explains 40% of total variation
Component 2 explains 14% of total variation

H3K36me3 p = 0.0023
H3K4me2 p = 0.0023
H3K9me2 p = 0.9
H3K27me1 p = 0.75

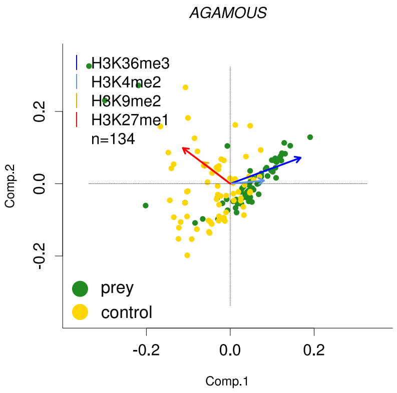

Component 1 explains 39% of total variation
Component 2 explains 17% of total variation

H3K36me3 p = 3.5e-06
H3K4me2 p = 0.00025
H3K9me2 p = 0.02
H3K27me1 p = 7.1e-05

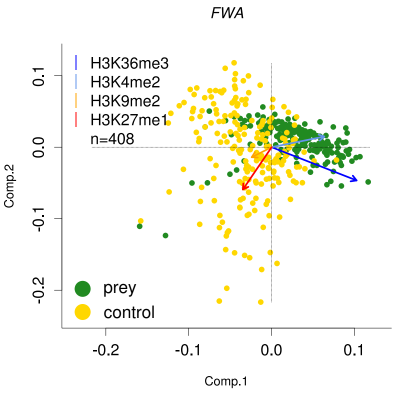

Component 1 explains 34% of total variation
Component 2 explains 13% of total variation

H3K36me3 p = 2e-16
H3K4me2 p = 8.8e-18
H3K9me2 p = 4.8e-09
H3K27me1 p = 0.00014

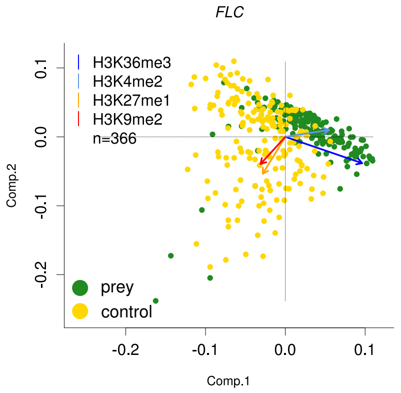

Component 1 explains 33% of total variation
Component 2 explains 14% of total variation

H3K36me3 p = 4.5e-16
H3K4me2 p = 3e-16
H3K27me1 p = 8e-06
H3K9me2 p = 2.3e-05
