# Supplementary material for: Characterization of chromosomal architecture in Arabidopsis by chromosome conformation capture
Source: Genome Biol. 2013 Nov 24;14(11):R129. doi: 10.1186/gb-2013-14-11-r129 (PMC4053840; doi:10.1186/gb-2013-14-11-r129)
Supplement: Additional file 17: Table S1 — Viewpoint coordinates and primer sequences. Indicated are the viewpoints’ names, their respective chromosome and position in bp, primer sequences, and restriction enzymes used for primary (1°RS) and secondary (2°RS) digest, respectively. Table S2. Alignment scores. Columns indicating chromosomes show numbers of mapped reads. Other columns show unmapped reads, percentage of mapped reads, and total reads. [file gb-2013-14-11-r129-S17.docx]

Additional File 1

**Additional File 1: Table S1, Viewpoint coordinates and primer sequences.**

Indicated are the viewpoints’ names, their respective chromosome and position in bp, primer sequences and restriction enzymes used for primary (1°RS) and secondary (2°RS) digest, respectively.

**Additional File 1: Table S2, Alignment scores.** Columns indicating chromosomes show numbers of mapped reads. Other columns: unmapped reads, percentage of mapped reads, and total of reads.

**Additional File 1: Table S1**

| **viewpoint** | **chromosome** | **position** | **Primer 1** | **Primer 2** | **1°RS** | **2°RS** |
| --- | --- | --- | --- | --- | --- | --- |
| *MEA F6* | Chr1 | 545880 | GAAGATGTACCATTATTACCAAGTG | TAAGTCAAACGATTGATGGTGTG | *Hind*III | *Nla*IV |
| *MEA F8* | Chr1 | 546537 | TCACCAGCAGTTCCATCATTC | GATAGACGTCATTGCCGTCG | *Hind*III | *Nla*IV |
| *AT1G51860* | Chr1 | 19260940 | TTTCTTTGACCTACAGGGAAGC | AGGAGTAACAAATACTTCTATGCATG | *Hind*III | *Nla*IV |
| *PHE* | Chr1 | 24265707 | GGCTGAGTATGTACATCAGGAGA | CCCCACGAAGTTAGTAGTTTCC | *Hind*III | *Dpn*II |
| *FIS2* | Chr2 | 14993834 | GGTCACGAGGTAGGCACTAA | GCCATAAACATTGAATTAGACTGTG | *Hind*III | *Dpn*II |
| *CKI1* | Chr2 | 19463781 | TGTATTGCAAAGGGGAAAGG | GTTTATGGTTTTCTTTGGTGGAA | *Hind*III | *Dpn*II |
| *AT3G44380* | Chr3 | 16035862 | CCTTAGCCGCTCCTTCAATA | CACAACCACGAAGTCACCAC | *Hind*III | *Nla*IV |
| *SWN* | Chr4 | 885827 | TTCGTTATTGGATGGTGAAGC | TGTAGTCCTCGGGACCAAGT | *Hind*III | *Nla*IV |
| *hk4s* | Chr4 | 1765891 | CCCGTGAACAAAACAGAGAAG | AATCCATCCTTGCATATTTGG | *Hind*III | *Nla*IV |
| *YAO* | Chr4 | 2745723 | AATTCCGAAAGCTCGATTCA | GGGCTCAAACCATAGACCAG | *Hind*III | *Nla*IV |
| *AG* | Chr4 | 10384033 | CGTAGAAATGGTTTGCTCAAGA | GACGATTCGTTGTGTTCTCG | *Hind*III | *Dpn*II |
| *FWA* | Chr4 | 13043163 | CTCCTGCTGCAAAGACCAC | AACATTATAGCAACTGCAAGGTCT | *Hind*III | *Dpn*II |
| *FLC* | Chr5 | 3178924 | CAAACACAGAACCGAGAAACAA | GGAAGAACAATGTCGTGAAGAA | *Hind*III | *Dpn*II |

**Additional File 1: Table S2**

|  |  |  |
| --- | --- | --- |
| **sample:** | **Reads with unique alignement** | **Reads with multiple alignements** |
| MEAF6_A | 2491932 | 189479 |
| MEAF6_B | 2507242 | 169389 |
| MEAF8_A | 2815594 | 93190 |
| MEAF8_B | 1981749 | 94675 |
| CKI1_A | 5231501 | 452827 |
| CKI1_B | 5639179 | 450389 |
| FLC_A | 6944301 | 698573 |
| FLC_B | 3222317 | 277053 |
| AG_A | 2748740 | 150224 |
| AG_B | 2831628 | 119835 |
| FIS_A | 3095292 | 219035 |
| FIS_B | 1242425 | 82548 |
| FWA_A | 7173457 | 644136 |
| FWA_B | 7179154 | 650431 |
| PHE1_A | 3705977 | 466211 |
| PHE1_B | 5647016 | 646799 |
| SWN_A | 2397897 | 200202 |
| SWN_B | 2258643 | 354821 |
| hk4s_A | 12045510 | 1935458 |
| hk4s_B | 9951888 | 1768489 |
| YAO_A | 5651474 | 244894 |
| YAO_B | 8242546 | 397882 |
| AT3G44380_A | 799168 | 88092 |
| AT3G44380_B | 1932529 | 253803 |
| AT1G51860_A | 3187836 | 163579 |
| AT1G51860_B | 6523009 | 270699 |
